# Increasing risk of vapor pressure deficit exceeding critical thresholds for tree growth

**DOI:** 10.1073/pnas.2530363123

**Published:** 2026-09-08

**Authors:** Tiewei Li, Hans W. Chen, Deliang Chen, Jingfeng Xiao, Lanlan Guo, Lianyou Liu, Feng Shi, Wenping Yuan, Ziqian Zhong, Huan Zheng, Bin He

**Affiliations:** ^a^ Department of Earth System Science, Tsinghua University, Beijing 100084, China; ^b^ Department of Environmental and Energy Sciences, Chalmers University of Technology, Gothenburg SE-412 96, Sweden; ^c^ Institute for Carbon Neutrality, Tsinghua University, Beijing 100084, China; ^d^Earth Systems Research Center, Institute for the Study of Earth, Oceans, and Space, University of New Hampshire, Durham, NH 03824; ^e^ Faculty of Geographical Science, Beijing Normal University, Beijing 100875, China; ^f^ State Key Laboratory of Lithospheric and Environmental Coevolution, Institute of Geology and Geophysics, Chinese Academy of Sciences, Beijing 100029, China; ^g^ Institute of Carbon Neutrality, Sino-French Institute for Earth System Science, College of Urban and Environmental Sciences, Peking University, Beijing 100871, China

**Keywords:** vapor pressure deficit, threshold, tree growth, expose, exceedance

## Abstract

With global warming, rising atmospheric vapor pressure deficit (VPD) has emerged as a critical driver of vegetation productivity and the terrestrial carbon sink. Yet, the thresholds beyond which VPD constrains tree growth remain poorly quantified. Using 2,953 tree-ring sites and combining generalized additive models with threshold regression models, we demonstrate that growth-related VPD thresholds are widespread. Approximately 63% of the studied species groups exhibited identifiable thresholds, which were generally lower in cool, humid regions and higher in warm, dry environments. For the same tree species occurring under contrasting aridity conditions, thresholds also differed substantially. As VPD has risen, the proportion of sites exceeding these thresholds has increased rapidly since about 1970 and is projected to rise further under different emissions scenarios. Under SSP5-8.5 in particular, approximately 79.8% of the sites are projected to be exposed to VPD above their thresholds by the end of the century. Although potential upward threshold shifts associated with acclimation or adaptation could partly reduce future exceedance, this buffering effect becomes increasingly limited under stronger climate forcing. Without effective climate mitigation, rising VPD will increasingly exceed the tolerance thresholds of trees, placing more trees under sustained growth constraints and undermining terrestrial carbon sequestration.

## Significance

Rising atmospheric vapor pressure deficit (VPD), a measure of atmospheric dryness, is increasingly recognized as a major threat to terrestrial ecosystems. Using global tree-ring records, we quantify growth-related VPD thresholds beyond which VPD-driven growth benefits weaken or VPD becomes inhibitory. As VPD continues to rise, more tree-ring sites are projected to exceed these thresholds. Although potential acclimation or adaptation may partly reduce this exposure, this buffering effect diminishes under stronger climate forcing, increasing the risk of widespread growth limitation and weakening Earth’s carbon sink.

Forests play a critical role in regulating the global carbon cycle by sequestering approximately 15% of anthropogenic CO_2_ emissions annually through photosynthesis and wood formation (1). As countries strive to meet emissions-reduction targets, forest ecosystems are increasingly relied upon for climate change mitigation through carbon sequestration (2). However, trees are becoming increasingly vulnerable to climatic extremes (3). Recent increases in heat stress and atmospheric aridity have been linked to widespread tree growth reductions and episodic, large-scale tree mortality across biomes, undermining forest carbon sinks (4–6). Clarifying the impacts of these climatic factors on tree growth is critical for assessing the vulnerability of forest ecosystems under ongoing warming.

Since the late 1990s, atmospheric vapor pressure deficit (VPD, defined as the difference between saturated and actual vapor pressure) has risen sharply (7) and has been identified as an increasingly important driver of plant functioning in terrestrial biomes (8–10). Elevated VPD intensifies evaporative demand, accelerating water loss from leaves and soils (11, 12). In response, trees often close their stomata to conserve water (13, 14), a strategy that reduces water loss but simultaneously limits carbon uptake, thereby suppressing photosynthesis and ultimately reducing tree growth and vegetation productivity (7, 15).

In contrast, some studies suggest that vegetation productivity may respond positively to increased VPD, particularly in humid and cool regions (16, 17). These divergent responses may depend on VPD magnitude, with negative effects emerging when VPD surpasses a critical threshold. For instance, a growing-season mean threshold of 0.35 to 0.40 kPa has been found to determine the direction of VPD effects on vegetation productivity in the Northern Hemisphere (17). However, due to differences in water-use strategies or stomatal regulation capacities among tree species, VPD thresholds may vary considerably (18, 19). To date, their variation among species and across climatic settings remains poorly quantified. It also remains unclear how rapidly these thresholds are being exceeded and how exceedance risk will evolve under future emissions. These gaps limit our ability to identify where and when atmospheric drying will constrain tree growth and to assess the risks to forest carbon-sink stability under continued warming.

Here, we used tree-ring data from 2,953 sites distributed primarily across North America, Europe, and western and central Asia to calculate site-level standardized ring-width indices as indicators of tree growth. To reduce the mixing of contrasting climate–growth relationships within some widely distributed species, we grouped tree-ring sites into 76 species groups, each comprising sites of the same species under comparable geographic and climate conditions. We then employed generalized additive models to explore nonlinear relationships between tree growth and VPD and used threshold regression models to identify the critical VPD thresholds for growth. We further examined the spatial patterns of these thresholds and their variation across climate gradients. Finally, we assessed historical and future changes in the proportion of sites experiencing VPD threshold exceedance and evaluated whether potential threshold shifts associated with acclimation could buffer future exceedance risk. By identifying growth-related thresholds, characterizing their spatial variation, tracking exceedance trajectories, and testing acclimation sensitivity, our study clarifies the critical role of VPD in governing forest vulnerability and resilience in a warming and drying world.

## Results

### Widespread Existence of VPD Thresholds for Tree Growth.

**Fig. 1.**
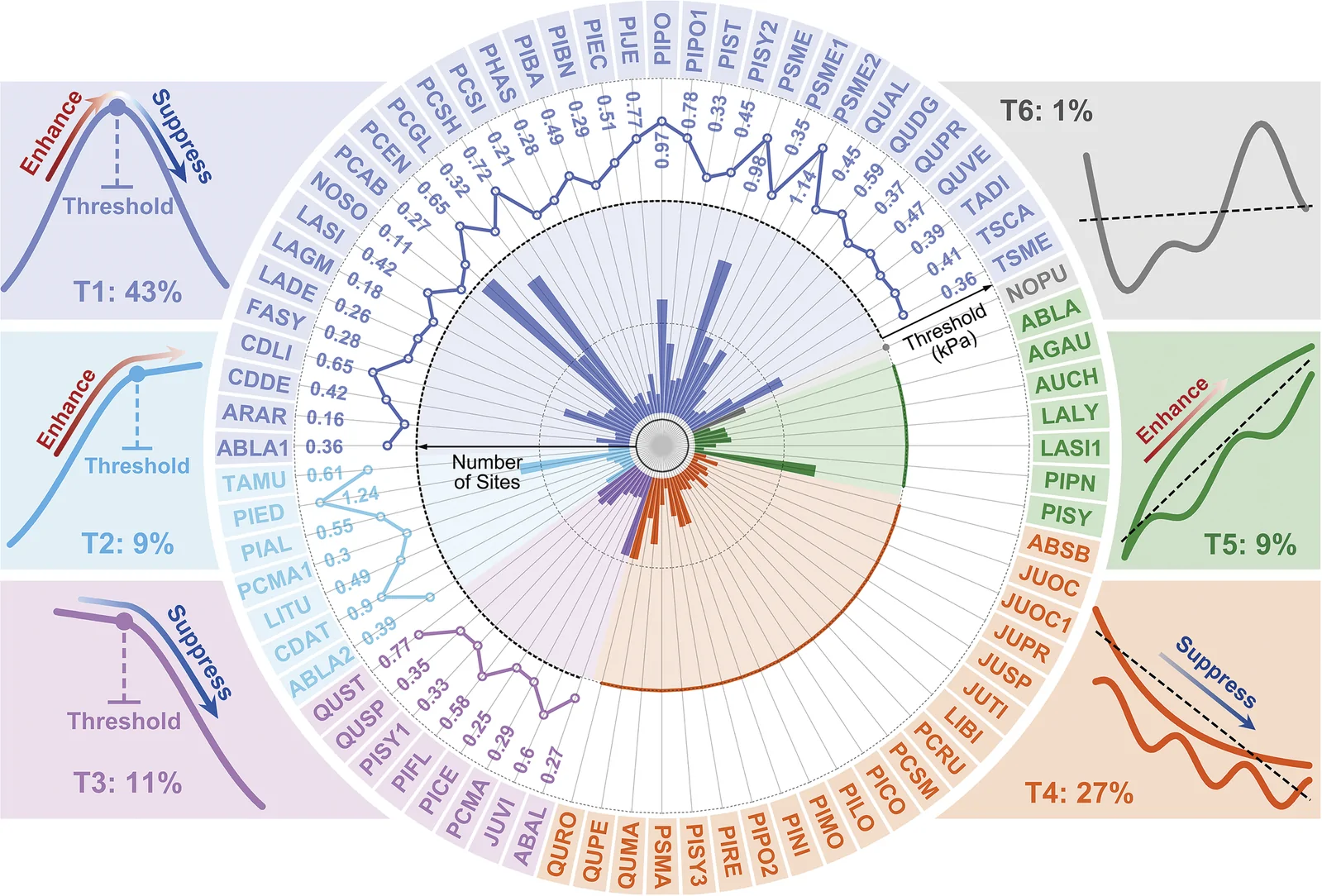
Critical VPD threshold affecting tree growth. Species-group codes, VPD thresholds, and numbers of tree-ring sites included in each species group are shown in the circular plot. The outer ring shows the species group codes, the middle line graph shows the VPD thresholds for each group, and the inner bars show the corresponding numbers of sites. The surrounding panels show the six typical response types of tree growth to VPD (T1 − T6).

We constructed multiple generalized additive models (GAMs) to examine the nonlinear effect of VPD on tree growth during the growing season for each species group (*Materials and Methods*). Our analysis revealed that the nonlinear effect of VPD on tree growth was widespread and could be classified into six types (T1–T6; Fig. 1 and *SI Appendix*, Fig. S1). Type T1 indicates that increasing VPD initially promotes tree growth but subsequently inhibits it; T2 shows that the promotional effect diminishes as VPD increases; and T3 exhibits an emerging inhibitory effect with rising VPD. Types T4 and T5 represent monotonic negative and positive effects, respectively, while T6 includes other response patterns that do not meet the criteria for T1–T5. Across the 76 species groups, including the same species inhabiting different aridity conditions, approximately 43% exhibited T1, while 9% and 11% exhibited T2 and T3, respectively. VPD exerted a monotonic negative effect on tree growth in approximately 27% of groups (T4) and a monotonic positive effect in 9% (T5). Only one species group (*NOPU*) showed irregular changes in response to increased VPD (*SI Appendix*, Fig. S1 and Table S1).

The response types T1, T2, and T3, comprising 63% of the tree species groups, suggest the presence of a critical VPD threshold. We used threshold regression models (TRMs) to identify the specific VPD thresholds for species groups exhibiting T1 − T3 (*Materials and Methods*; Fig. 1 and *SI Appendix*, Figs. S2 and S3). For example, in *Picea abies* (PCAB), which exhibited a T1 response, the models identified a VPD threshold of 0.27 kPa (0.25 to 0.32 kPa, 95% CI; *SI Appendix*, Fig. S3*A*). For *Liriodendron tulipifera L.* (LITU) and *Juniperus virginiana L.* (JUVI), which exhibited T2 and T3 responses, threshold regression identified VPD thresholds of 0.49 kPa (0.39 to 0.50 kPa, 95% CI) and 0.60 kPa (0.59 to 0.64 kPa, 95% CI), respectively (*SI Appendix*, Fig. S3 *C* and *E* and Table S1). For response types characterized by monotonic negative (T4), positive (T5), or irregular (T6), we did not estimate thresholds for these species groups to avoid the risk of identifying spurious thresholds (*SI Appendix*, Fig. S3 *B*, *D*, and *F*). To assess robustness, we also extracted GAM-derived thresholds for T1 − T3 by identifying the turning point at which the derivative of the VPD marginal effect curve reached or approached zero (20). The thresholds estimated by the two approaches were generally consistent. The differences between TRM-derived and GAM-derived thresholds were within ±0.1 kPa, with a mean absolute difference of 0.03 kPa (*SI Appendix*, Fig. S4).

**Fig. 2.**
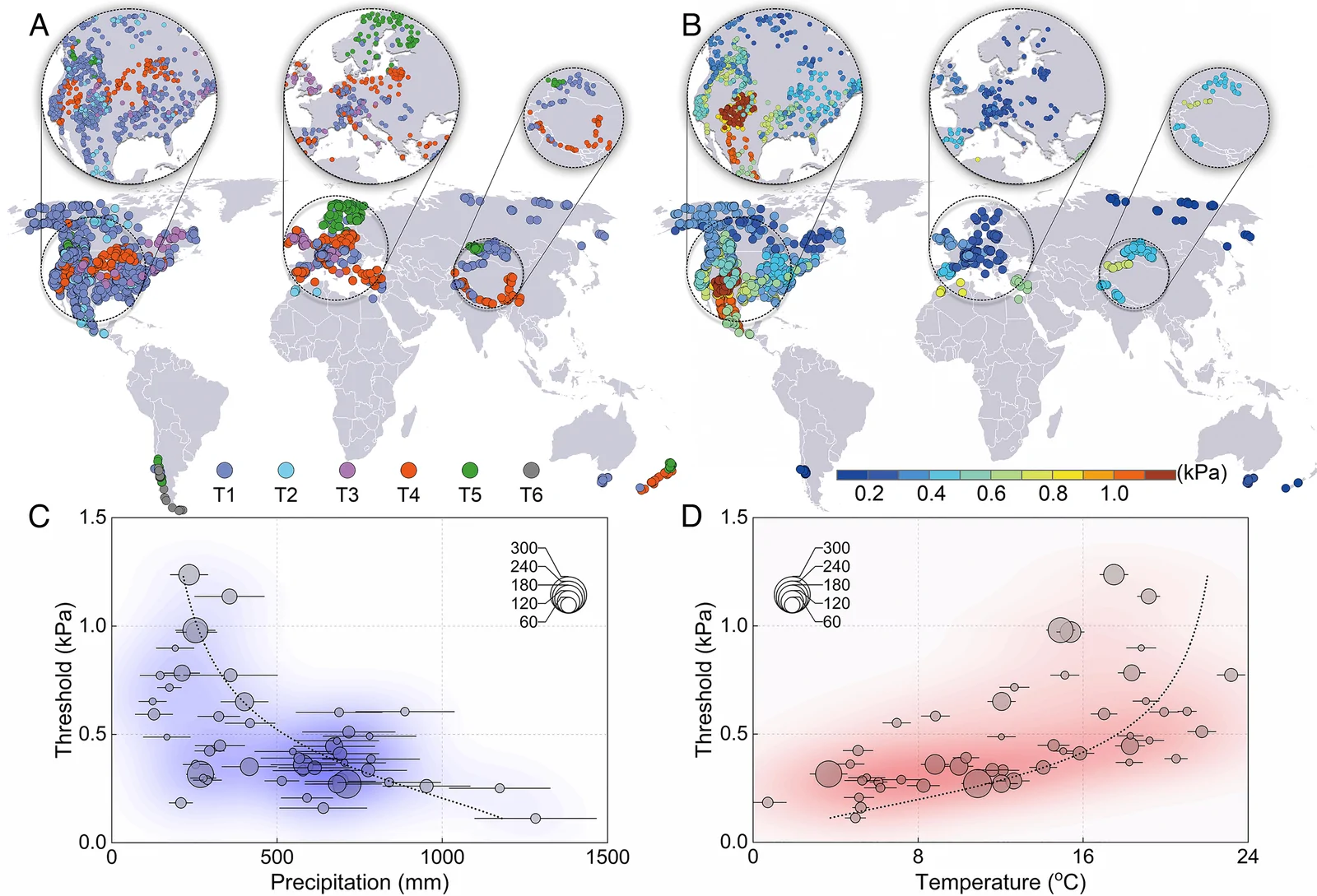
Spatial patterns of the six response types and thresholds. (*A*) Spatial distribution of the six types of tree growth responses to VPD based on generalized additive models. (*B*) Spatial distribution of the critical VPD threshold based on threshold regression models. (*C* and *D*) Variation in thresholds along precipitation (*C*) and temperature (*D*) gradients. Circle size indicates the number of sites included in each species group. The filled contours (blue: precipitation; red: temperature) indicate the density distribution of the points. Error bars indicate the SE of precipitation and temperature.

We further assessed the spatial distribution of the six types and corresponding VPD thresholds (Fig. 2 *A* and *B*). T1 is widely distributed in North America, Central Europe, and Asia. T2 is mainly distributed in a few regions of North America, such as northern Canada, the Rocky Mountains, and Mexico. T3 is mainly found in the eastern United States and the United Kingdom. T4 is mainly distributed in the western and northern United States, Central Europe, the Mediterranean region, and the Qinghai-Tibet Plateau. T5 is mainly found in high-latitude regions such as Sweden and Finland. Generally, low VPD thresholds are prevalent in most cold and humid regions, such as high-latitude and high-altitude regions, while high thresholds are mainly observed in drier and hotter regions (Fig. 2 *C* and *D*). For example, tree species inhabiting arid regions of the western United States show relatively high thresholds affecting their growth (Fig. 2*B*). Importantly, even for the same tree species occurring under different aridity conditions, the threshold affecting their growth can vary significantly. For instance, the threshold for *Pseudotsuga menziesii* (PSME) in arid regions (mean Aridity Index (AI) = 0.28) is 0.98 kPa, compared with 0.35 kPa in humid regions (mean AI = 1.34). A similar phenomenon was observed in *Pinus ponderosa* (PIPO) and *Pinus sylvestris L.* (PISY) (*SI Appendix*, Fig. S5 and Table S1).

### Warming Increases the Proportion of Sites Exceeding VPD Thresholds.

**Fig. 3.**
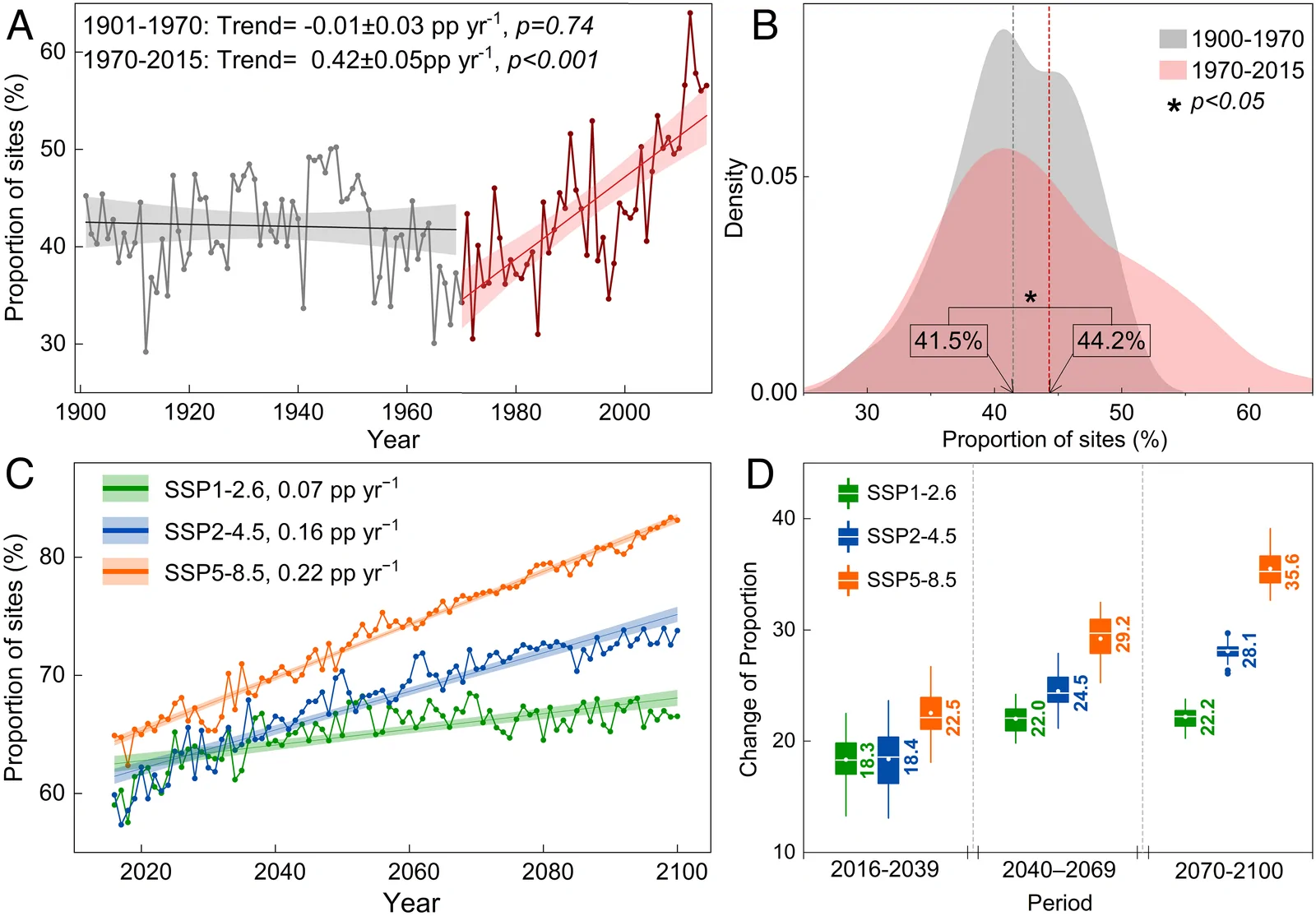
Historical and projected trends in the proportion of sites exceeding VPD thresholds. (*A*) Historical temporal change in the proportion of sites exceeding VPD thresholds during 1901−2015. Shaded areas indicate the 95% CI. (*B*) Significant increase in the proportion of sites exceeding thresholds between the 1901–1969 and 1970–2015 periods (*t* test, *P* < 0.05). (*C*) Projected temporal change in the proportion under three future emissions scenarios. Trends were estimated using linear regression, and the shaded areas around the regression lines represent 95% CI. (*D*) Projected changes in exceedance proportion compared with the historical period (1970–2015) across different future time periods. For each boxplot, the *Bottom*, *Middle*, and *Top* of the box are the 25th, 50th, and 75th percentiles, respectively, and the *Bottom* and *Top* whiskers show the 10th and 90th percentiles, respectively. The white point in each box represents the mean proportion change, with corresponding values labeled to the *Right* of each box.

With the intensification of global warming, the proportion of tree-ring sites exceeding VPD thresholds showed a sharp increase after about 1970 (trend = 0.42 ± 0.05 percentage points per year (pp y^−1^), *P* < 0.001, Fig. 3*A*), whereas no significant change occurred before 1970 (trend = − 0.01 ± 0.03 pp y^−1^, *P* = 0.74). During 1970–2015, the average proportion of sites exceeding thresholds was 44.2%, significantly higher than the 41.5% observed during 1901–1969 (*P* < 0.05; Fig. 3*B*). Simulations from Earth system models in the Coupled Model Intercomparison Project Phase 6 (CMIP6) indicate that the proportion of sites exceeding VPD thresholds will continue to increase from 2016 to 2100 (Fig. 3*C*). Under a low-emissions scenario (SSP1-2.6) and a moderate-emissions scenario (SSP2-4.5), the proportion increases moderately by 0.07 pp y^−1^ and 0.16 pp y^−1^, respectively. However, under a high-emissions scenario (SSP5-8.5), the proportion increases quickly by 0.22 pp y^−1^. This rising trend in threshold exceedance suggests that more trees are being exposed to high growing-season VPD levels beyond their physiological tolerance limits.

The differences between the historical exceedance proportion (1970–2015) and projected proportions under the three scenarios are similar in the near future (2016–2039), with mean increases of 18.3, 18.4, and 22.5 percentage points under SSP1-2.6, SSP2-4.5, and SSP5-8.5, respectively. However, these differences widen by mid-century (2040–2069) and further toward the end of the century (2070–2100). By the end of the century, the exceedance proportion under SSP5-8.5 substantially outpaces that of the historical period, with a mean increase of 35.6 percentage points (Fig. 3*D*) and approximately 79.8% of sites projected to experience VPD conditions exceeding their thresholds. This temporal distinction in projected proportions under the three scenarios underscores that, before mid-century, adopting strategies to mitigate warming could reduce the risk of exceeding VPD thresholds by the end of the century.

### Potential Threshold Shifts Only Partly Buffer Future VPD Exceedance Risk.

The climate projections above assume that the VPD thresholds estimated from historical growth responses remain stationary into the future. However, these projected threshold-exceedance proportions may be modulated by the potential capacity of trees to acclimate to sustained atmospheric dryness. For instance, experimental studies have shown that acclimation to elevated VPD can modify stomatal regulation, maintain carbon gain under higher VPD, and alter traits associated with hydraulic vulnerability (21, 22). Such physiological adjustments may shift the VPD level at which growth becomes constrained. Consequently, if such acclimation or adaptation occurs, VPD tolerance thresholds may shift upward, potentially buffering some of the projected risks.

**Fig. 4.**
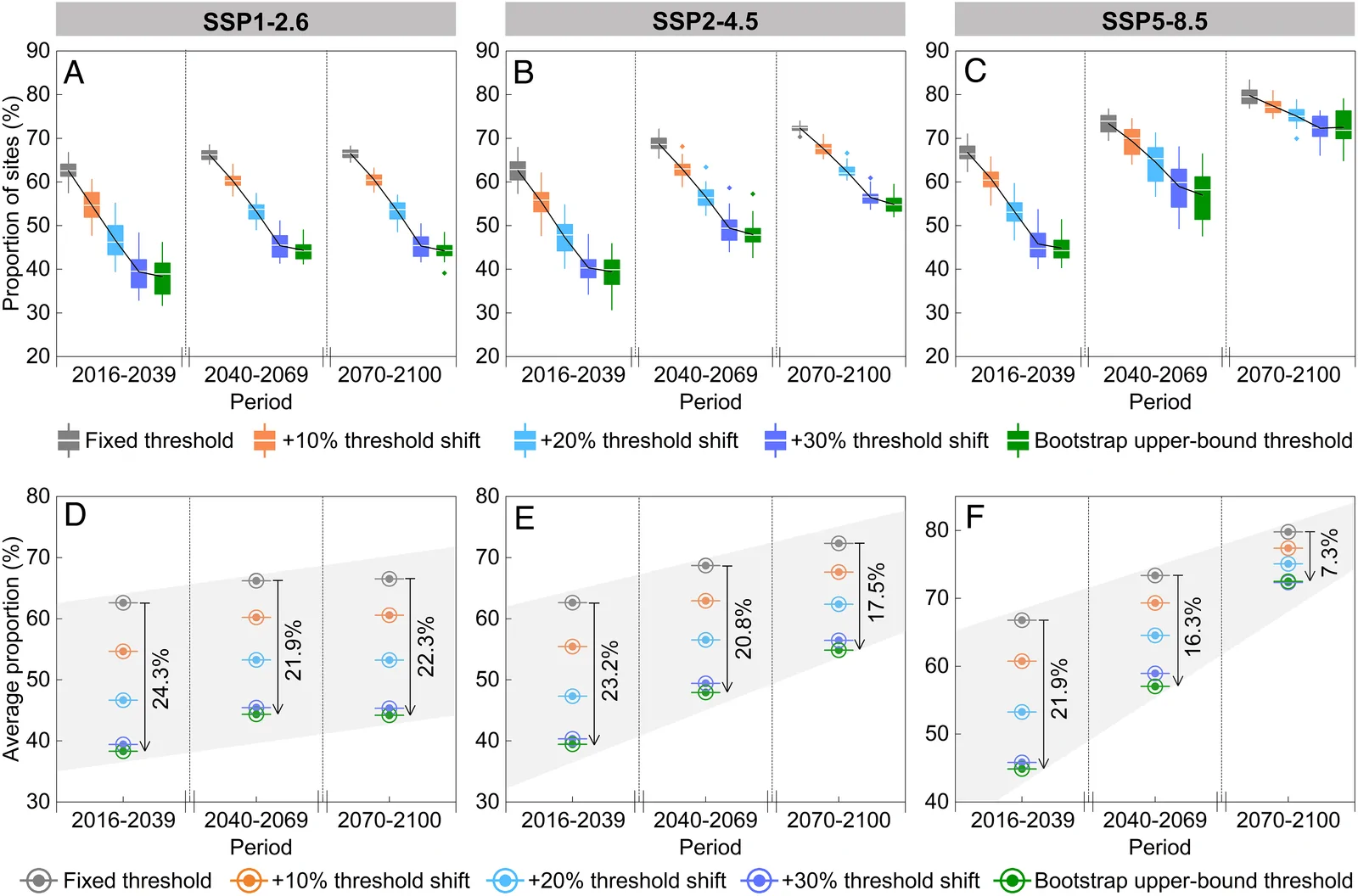
Sensitivity analysis of future changes in the proportion of sites exceeding VPD thresholds. (*A*–*C*) Distributions of the proportion of sites exceeding thresholds across time periods (2016−2039, 2040−2069, 2070−2100) under alternative threshold-shift assumptions, shown as boxplots. The fixed-threshold baseline assumes that VPD thresholds estimated from historical tree-growth responses remain unchanged in the future. The +10%, +20%, and +30% threshold-shift scenarios represent hypothetical upward shifts in thresholds that could arise from potential acclimation or adaptation to increasing atmospheric dryness. The bootstrap upper-bound threshold represents a high-threshold case based on the upper confidence bound of the threshold estimates. (*D*–*F*) Mean exceedance proportions under each threshold assumption across the three future periods. Arrows indicate the reduction in mean exceedance proportion between the fixed-threshold baseline and the bootstrap upper-bound threshold.

To account for this potential acclimation of tree species to VPD, we conducted a sensitivity analysis of the future proportion of exceedance. We recalculated future exceedance proportions under five threshold assumptions: a fixed-threshold baseline, three scenarios with relative threshold increases of +10%, +20%, and +30%, and a bootstrap upper-bound threshold case (*Materials and Methods*). We found that simulating threshold increases (as a proxy for acclimation) consistently reduced the projected exceedance proportion, but the magnitude of this reduction depends strongly on emissions forcing and time (Fig. 4). Under SSP1-2.6, progressively larger upward threshold shifts consistently reduced the proportion of sites exceeding VPD thresholds, with broadly comparable reductions across the three future periods (Fig. 4*A*). The mean exceedance proportion was reduced by 24.3, 21.9, and 22.3 percentage points across 2016–2039, 2040–2069, and 2070–2100 relative to the fixed-threshold baseline, respectively (Fig. 4*D*). However, under SSP2-4.5, the divergence among threshold-shift scenarios progressively narrows toward the end of the century (Fig. 4*B*), with reductions declining from 23.2 to 20.8 and 17.5 percentage points across the same periods (Fig. 4*E*). Under SSP5-8.5, this convergence is even more pronounced (Fig. 4*C*). The reduction drops from 21.9 to 16.3 and then to only 7.3 percentage points by 2070–2100 (Fig. 4*F*), indicating that threshold increases provide progressively less protection against exceedance risk under stronger forcing. Importantly, even under the higher threshold shift scenario (+30%) and bootstrap upper-bound threshold case, end-of-century exceedance remains critically high under SSP5-8.5 (72.3% and 72.5%, respectively). This finding suggests that potential threshold shifts associated with acclimation or adaptation alone are unlikely to fully counteract projected increases in exceedance risk under high emissions and further underscores that, without effective mitigation efforts, rising VPD will increasingly exceed the tolerance thresholds of trees, posing a severe threat to their growth.

## Discussion

In this study, we demonstrate that tree growth exhibits widespread nonlinear responses to VPD and identify critical thresholds affecting growth. The thresholds identified here mark the level of growing-season VPD at which the positive effect begins to weaken or even become negative. The existence of these thresholds is likely linked to the cumulative effect of stomatal regulation, whereby trees adjust stomatal conductance to optimize the trade-off between carbon gain and water loss (23). When VPD remains below a threshold, moderate increases in atmospheric demand coincide with relatively open stomata and enhanced photosynthetic rates (17, 24, 25), and may also benefit tree growth through other pathways, such as by alleviating soil hydromorphy (26). However, as VPD rises toward and beyond a threshold, trees increasingly downregulate stomatal conductance to prevent dangerous declines in leaf water potential (27, 28). This protective behavior comes at the cost of reduced carbon assimilation and allocation to growth (29). Consequently, this physiological regulation drives the initially positive effect of VPD to weaken progressively and further become inhibitory, yielding the threshold responses observed in types T1–T3.

The monotonic response types (T4 and T5) do not necessarily imply that thresholds or significant responses are absent; rather, they likely reflect the limited range of historical VPD observations relative to the species’ physiological niche. If a threshold lies outside the observed VPD range, the data capture only one segment of the response curve, resulting in an apparently monotonic relationship. For instance, T4 may arise when observations fall largely within the postthreshold range, whereas T5 may occur when observations remain on the prethreshold side (Fig. 2*A* and *SI Appendix*, Fig. S6). If VPD continues to increase, it could progressively push these species toward threshold exceedance, such that species currently classified as T5 may eventually shift toward one of the threshold-like response patterns.

VPD thresholds for growth vary widely among species and are notably higher in arid, hot regions than in humid, cool regions (Fig. 2). This spatial pattern aligns with interspecific differences in stomatal sensitivity to rising VPD, which determine the timing of stomatal closure (18, 30, 31). Species in humid climates often exhibit higher stomatal sensitivity than those in arid climates (32–34), implying earlier downregulation of conductance and carbon assimilation and thus lower VPD thresholds. Conversely, drought-adapted species tend to maintain gas exchange at higher VPD levels, delaying carbon limitation and yielding higher thresholds. Thresholds also varied within species across contrasting climates, potentially reflecting acclimation and adaptation to local conditions. Experimental studies show that sustained exposure to elevated VPD can modify stomatal regulation and water-use traits (21, 22), and that populations of the same species originating from contrasting climates can differ in drought tolerance, xylem structure, and stomatal responsiveness (35, 36). Therefore, interspecific and intraspecific variation in thresholds is likely underpinned by a suite of functional traits (37, 38), such as shifts along the iso/anisohydric continuum (39–41), more negative leaf turgor loss points (42, 43), and larger hydraulic safety margins or greater resistance to xylem embolism (28, 44, 45). Linking thresholds to functional traits, population differentiation, and genetic variation may clarify which physiological and structural mechanisms govern both the magnitude of thresholds and their plasticity across climates.

The proportion of sites exceeding VPD thresholds increased rapidly after about 1970 and is projected to continue rising under all emission scenarios, especially under SSP5-8.5. This increase means that more trees will be exposed to VPD conditions under which further atmospheric drying increasingly constrains radial growth. As such exposure becomes more common, growth suppression may recur across years, potentially leaving persistent legacy effects and gradually reducing the capacity of trees to resist and recover from subsequent stress (46–48). Such persistent growth declines and losses of growth resilience have been reported to be associated with elevated mortality risk under drought and heat stress (49–51). We also found that VPD above the corresponding growth-related threshold was common among our matched mortality event–species records (*SI Appendix*, Fig. S7). However, this observed co-occurrence does not yet permit robust inference about the relationship between threshold exceedance and mortality, because the dataset used here was restricted to documented heat- and drought-related die-off events and has limited spatial representativeness (52). More extensive and standardized long-term monitoring data on tree mortality (53) will be needed to quantify this relationship more reliably and to evaluate whether incorporating threshold exceedance can improve the accuracy of tree-mortality predictions under continued warming (54, 55).

The future extent of threshold exceedance will also depend on whether trees can adjust their responses as atmospheric dryness intensifies. Experimental studies show that prolonged exposure to elevated VPD can shift the VPD optimum for transpiration and alter stomatal regulation and traits controlling water loss (21, 22). These adjustments could allow trees to maintain growth under drier conditions, raising the VPD level at which atmospheric dryness begins to constrain growth, potentially shifting thresholds upward. For instance, climate-vulnerability models produce lower projected risks when physiological limits, phenotypic plasticity, and evolutionary adaptation are allowed to vary rather than being held fixed (56). Our sensitivity analysis similarly showed that upward threshold shifts could buffer future exceedance risk. But in the absence of effective climate mitigation, this buffering effect declined in later periods because continued increases in VPD increasingly exceeded the protection provided by a finite upward shift in the threshold (Fig. 4). These findings underscore the reality that forest systems face finite limits to adaptation (57) and that relying solely on autonomous adaptation is unlikely to be sufficient to cope with future climate change (58). Accordingly, integrating trees’ intrinsic capacity for acclimation and adaptation with effective climate-mitigation practices represents a key strategy for sustaining the long-term stability of forest carbon sinks.

This sensitivity analysis is based on hypothetical scenarios (59, 60). In reality, the degree to which different species can acclimate to rising VPD and the resulting potential shifts in thresholds may be far more complex (61). Future work integrating long-term monitoring networks with manipulative experiments and tree-ring evidence is therefore needed to quantify how VPD tolerance thresholds systematically adjust over time. In addition, averaging VPD over the growing season may smooth high VPD episodes, and the thresholds identified here therefore reflect the cumulative influence of atmospheric dryness on tree growth. Future studies using photosynthesis proxies with higher temporal resolution could further identify VPD thresholds for short-term physiological processes, such as midday depression of photosynthesis (62), and clarify how these short-term responses scale up to annual growth limitation. Overall, our study provides timely and critical evidence that VPD constrains tree growth in a nonlinear, threshold-like manner and that these thresholds vary systematically across climate gradients. The climate projections further show that widespread threshold exceedance becomes increasingly difficult to avoid under higher emissions, lending urgency to mitigation efforts that limit atmospheric drying and preserve the long-term stability of forest carbon sinks (63, 64).

## Conclusions

Using tree-ring data and combining generalized additive models with threshold regression models, our study demonstrates that VPD thresholds for growth are widespread across species. These thresholds vary considerably, both among different species and within the same species across different environments. Future projections indicate that the proportion of sites exceeding thresholds will increase under all three scenarios, but substantially more so under the SSP5-8.5 scenario than under the other two. Although potential acclimation or adaptation could partly reduce future exceedance by shifting thresholds upward, this buffering effect becomes increasingly limited under stronger climate forcing. Without effective climate mitigation strategies, progressively more trees are projected to be exposed to VPD levels above their threshold by the end of the century, undermining the capacity of forests to act as a resilient carbon sink.

## Materials and Methods

### Tree-Ring Datasets.

Tree-ring width measurements provide information about tree radial growth and climatic signals (65, 66). The raw tree-ring width measurements were obtained from the International Tree Ring Data Bank (ITRDB). We applied the regional curve standardization method to remove the age-related trends from raw tree ring width measurements and preserve the effects of environmental signals at interannual timescales using the *dplR* package in R (67, 68). Raw ring-width series were aligned by cambial age, and the mean ring width across all available series was calculated for each cambial-age class. A smoothing spline with a 50% frequency-response cutoff at 10% of the maximum cambial-age wavelength was then fitted to this age-specific mean series to construct the Regional Curve. Tree-level standardized ring width indices were calculated as ratios between individual series and the smoothed Regional Curve (69). For tree-ring series from the Southern Hemisphere, the calendar year assigned to the ring was that during which ring formation started (70). Based on the availability of key metadata required for the study, such as longitude, latitude, elevation, species code, and tree-ring measurements, a total of 3,485 sites were selected. Multiple tree-level standardized tree-ring indices were processed into site-level standard chronologies using the biweight robust mean method (*SI Appendix*, Fig. S8) (71).

Because climate–growth relationships may vary with species identity and environmental context, we grouped tree-ring sites before threshold estimation to avoid combining potentially different growth responses (72–74). We first classified all tree-ring sites by species code. For geographically separated subsets of a species, subsets containing fewer than 10 sites were excluded before further grouping. This step removed a small number of geographically isolated sites and ensured that the retained sites of each species were relatively geographically coherent before further climate-based grouping. For example, 279 *Pseudotsuga menziesii* sites were located in North America and only three sites were located in Europe. The three European sites were excluded from subsequent grouping (*SI Appendix*, Fig. S9). We then classified the retained sites within each species according to geographic location and environmental context, including long-term mean annual temperature, long-term mean annual precipitation, and aridity index. Species occurring across clearly different climate environments were divided into separate species groups. For example, *Pseudotsuga menziesii* was divided into PSME, PSME1, and PSME2, with mean aridity indices of 0.28, 1.34, and 0.45, respectively (*SI Appendix*, Figs. S10 and S11). In some cases, sites were geographically dispersed but occurred under broadly similar climate conditions. These sites were retained as a single species group. For example, although *Larix gmelinii* sites were geographically dispersed, most occurred in cool and humid environments in high-latitude of Russia. After grouping, 2,953 tree-ring sites representing 76 species groups were retained (*SI Appendix*, Fig. S12).

### Climate Datasets.

Monthly mean, maximum and minimum temperature, precipitation, actual vapor pressure, and potential evapotranspiration data from 1901–2015 were sourced from the Climate Research Unit dataset provided by the University of East Anglia in the UK (CRU TS 4.07, 0.5° × 0.5°). Maximum and minimum temperature and actual vapor pressure were used to calculate the vapor pressure deficit (VPD) (75, 76):

[1] $$e^{0}\left(T\right)=0.6108\;exp\left[\frac{17.27T}{T+237.3}\right],$$

[2] $$e_{s}=\frac{e^{0}\left(T_{\max}\right)+e^{0}\left(T_{\min}\right)}{2},$$

[3] $$\mathrm{VPD}=e_{s}-e_{a},$$

where T is the air temperature (°C) and e^0^(T) is the saturated vapor pressure at temperature T (kPa); e_s_ is the saturated vapor pressure; T_max_ and T_min_ are monthly maximum and minimum temperatures; and e_a_ is the actual vapor pressure. Precipitation and potential evapotranspiration datasets were utilized to compute the aridity index (AI), defined as the ratio of mean annual precipitation to mean annual potential evapotranspiration. This index facilitated the identification of arid (AI < 0.5) and humid (AI ≥ 0.65) regions, with a lower AI indicating drier climatic conditions (77, 78). The monthly shortwave radiation data (0.5° × 0.5°; 1901−2012) were obtained from the Terrestrial Hydrology Research Group at Princeton University (79). All monthly climate variables used in this study were aggregated over growing seasons from April to October for Northern Hemisphere sites and October to March of the following year for Southern Hemisphere sites to match the seasonal window used for tree-ring growth responses (10, 80, 81). Finally, we combined annual growing season mean temperature, total precipitation, mean shortwave radiation, and mean VPD calculated above with the chronologies to examine nonlinear VPD–growth relationships and identify VPD thresholds. Detailed information on the datasets used in this study is provided in *SI Appendix*, Table S2.

The climate factors used to project changes in the proportion of sites exceeding VPD thresholds were generated from Coupled Model Intercomparison Project phase 6 (CMIP6) models (82). Compared with CMIP5, future CMIP6 scenarios combine Shared Socioeconomic Pathways with representative concentration pathways (83, 84). For example, SSP1−2.6 represents the future scenario incorporating SSP1−based socioeconomic development into the representative concentration pathway 2.6-based energy-emissions-land-use scenarios (85). Detailed information on the datasets used in this study is provided in *SI Appendix*, Table S3. We used monthly T_max_, T_min_, and relative humidity data from eleven Earth system models under the SSP1–2.6, SSP2–4.5 and SSP5–8.5 scenarios, which represent low-, medium-, and high-emissions pathways, respectively (*SI Appendix*, Table S3). Relative humidity (RH) data were utilized to calculate the actual vapor pressure (76):

[4] $$e_{a}=e_{s}\times \mathrm{RH}.$$

We used 2015/2016 as the boundary between the observation-based historical analysis and model-based future analysis because CMIP6 ScenarioMIP simulations begin in 2015. To avoid overlap between the historical analysis and the scenario-based projections, historical exceedance was summarized through 2015, whereas projected exceedance was analyzed from 2016 onward.

### Identification of the Nonlinear Effects of VPD on Tree Growth.

We used generalized additive models (GAMs) to explore the nonlinear effects of VPD on tree growth (86). The generalized additive model is a statistical model used for modeling nonlinear relationships and it relaxes the requirements for the linear conditions of the model, which can be applied to arbitrarily distributed data, and can reasonably find the nonmonotonic and nonlinear relationship between the explanatory variables and the response variables. The generalized additive model fits the variables by multiple smooth functions, such as smoothing spline function, kernel function, or local regression smooth function (*SI Appendix*, Fig. S13):

[5] $$g\left(y\right)=\beta_{0}+\sum_{i=1}^{n}f_{i}\left(x_{i}\right)+\varepsilon ,$$

where *g* is the link function of the expected variable *y* and the explanatory variables *x_i_* for each species group; the function *f_i_* is a smooth function with specified parameter forms; *β_0_* is an intercept term; ε is residual error; and *n* is the number of smooth functions (87). We used the restricted maximum likelihood smoothing parameter estimation method to select appropriate model parameters to improve the prediction accuracy and reliability of the model, as it is less prone to undersmoothing and is unlikely to develop multiple minima (88, 89). We used marginal effect plots to explore the response of tree growth to VPD. The marginal effect plots visually show the individual component effects of each smooth term, while keeping other factors in the model constant and focusing only on the effect of VPD on tree growth (90).

In this study, we included four climate variables (average temperature, shortwave radiation, VPD, and total precipitation during the growing season), a time variable (year), and three location variables (latitude, longitude, and altitude). We lumped the data from all sites within each species group, using standardized chronologies as response variables and climatic, time, and location variables as explanatory variables. Response curves of tree growth to VPD were generated using GAMs. All GAMs were fitted using the years available for the tree-ring chronologies, whose temporal coverage fell within 1901–2012. We report the significance of the VPD smooth (*P* < 0.1) and the effective degrees of freedom (*edf*), where an *edf* close to 1 indicates an approximately linear effect and higher *edf* indicates stronger nonlinearity (*SI Appendix*, Table S1) (91).

To evaluate whether collinearity among climate drivers affects the inferred VPD response, we conducted a model sensitivity analysis for each species group. We fitted eight GAM models: (M1) a VPD-focused model; (M2 − M4) models that additionally included temperature, precipitation, or shortwave radiation individually; (M5) a full additive model including all climate predictors; and (M6 − M8) models that further included tensor interaction terms between VPD and temperature, precipitation, or shortwave radiation to allow the VPD effect to vary with background climate conditions (92). We then compared the marginal-effect curves of VPD across the eight models. For each species group, we used the Akaike information criterion (AIC) to identify the best GAM among the eight candidate models. We considered the VPD response to be robust when its overall shape remained stable across the eight models and agreed with the AIC-selected best model, and when any identified threshold occurred within the observed VPD range. Across the 76 species groups, 60.5% and 36.8% exhibited consistent VPD marginal-effect curves across all eight models and across seven models, respectively (*SI Appendix*, Fig. S14). This sensitivity analysis indicates that the nonlinear VPD–growth relationships are robust and not driven by collinearity among climate predictors.

### Identification of VPD Thresholds.

We used threshold regression models (TRMs) to identify the critical VPD thresholds for tree growth. TRMs are a class of regression models in which predictors are associated with the outcome in a threshold-dependent way, explicitly introducing a threshold parameter that allows thresholds to be quantified (93). In this study, we used the segmented threshold regression model:

[6] $$\eta =\alpha_{1}+\alpha_{2}^{T}z+\beta {\left(x-b\right)}_{+}+\gamma x,$$

[7] $${\left(x-b\right)}_{+}=\left\{\begin{array}{c} x-b,x>b\\ 0,x\leq b \end{array},\right.$$

where *b* is the threshold parameter, *x* is the predictor with a threshold effect, *z* denotes additional predictors, α,β,γ are model coefficients, the superscript *T* in $\alpha_{2}^{T}z$ denotes matrix transposition, and *η* represents the regression function of the model. The hinge function (*x* − *b*)_*+*_ equals *x* − *b* when *x* > *b* and 0 otherwise.

To reduce the risk of identifying spurious thresholds, we first classified the GAM curves. We fitted a segmented linear function to the VPD marginal-effect curve from the AIC-selected GAM for each species group and classified the response according to the direction and significance (*P* < 0.05) of the slopes before and after the breakpoint (94, 95) (*SI Appendix*, Table S4). Curves that first increased significantly and then decreased significantly were classified as T1. Curves that first increased significantly and then showed no significant change were classified as T2, whereas those that initially showed no significant change and then decreased significantly were classified as T3. Curves that first decreased significantly and then either continued to decrease significantly or showed no significant change were classified as T4 (e.g., JUSP). Curves that either increased significantly in both segments or initially showed no significant change and then increased significantly were classified as T5 (e.g., AUCH). Other response patterns were classified as T6. For example, NOPU showed a significant decrease followed by a significant increase.

For species groups classified as T1–T3, the threshold parameter was subsequently estimated from the original observations using the TRMs, and its 95% CI was obtained from 1,000 bootstrap replicates. This two-step procedure was used because GAMs are well suited to flexibly characterize the potentially nonlinear VPD–growth relationship and to diagnose whether a threshold-like response exists, but GAMs do not directly provide a parameterized threshold estimate. In contrast, TRMs explicitly estimate a threshold parameter and its uncertainty, but they can also return a threshold even when the underlying relationship is weakly nonlinear or linear, which may yield a spurious threshold (96). Therefore, we estimated an explicit threshold using TRMs only when the GAMs indicated a clear threshold-type response (T1 − T3). This two-step workflow prevents overidentifying thresholds while providing a more robust and quantifiable threshold estimate (*SI Appendix*, Fig. S15).

We also assessed whether the two approaches yielded similar threshold locations. Because GAMs do not directly provide a robust threshold estimate, we derived an approximate GAM-based turning point by identifying where the first derivative of the GAM-estimated VPD marginal effect reached or approached zero (20). We quantified uncertainty using 1,000 bootstrap replicates to obtain CI for the turning point. Derivative-based turning points can be sensitive to local curvature and typically have larger uncertainty, so the derivative-based turning points from GAMs are best viewed as an approximate diagnostic. Therefore, we relied on TRMs as the primary and more robust method for threshold estimation, while using the GAM-based turning points as an independent consistency check.

### Proportion of Sites Exceeding Thresholds.

The proportion of sites exceeding the estimated threshold was calculated as

[8] $$\mathrm{Prop}\left(X_{i}>\mathrm{Threshold}\right)=\frac{n\left(X_{i}>\mathrm{Threshold}\right)}{N\left(i\right)}\times 100\%,$$

where *X* is the historical or simulated VPD value under each future scenario; *n*() is the number of sites where VPD exceeds the threshold for each year (*i*); and *N*(*i*) is the number of sites belonging to species groups with identified thresholds in year *i*. The time slice of projected proportion includes near future (2016–2039), mid-century future (2040–2069), and end of century (2070–2100).

To account for the possibility that trees may acclimate to increasing VPD and thereby shift their tolerance thresholds upward, we conducted a sensitivity analysis of future exceedance proportions. Previous climate-vulnerability modeling studies have commonly used fixed-threshold projections as a baseline and then evaluated how projections change when physiological limits, adaptive capacity, or local adaptation are allowed to vary (56, 97, 98). When the parameters controlling adaptive responses are uncertain or unavailable for many species, sensitivity analyses provide a transparent way to evaluate whether projected vulnerability is robust to alternative assumptions about adaptive capacity (60).

Therefore, we used the fixed-threshold baseline as the no-acclimation scenario, in which thresholds estimated from historical observations were held fixed when calculating future exceedance proportions. We then evaluated three hypothetical relative threshold increases of +10%, +20%, and +30%. The selection of these shifts was informed by the bootstrap-derived upper bounds of the historical threshold estimates. Across species groups, the +30% scenario was generally close to the corresponding bootstrap-derived upper bound, whereas larger increases, such as +40% or +50%, would extend beyond the uncertainty range of the estimated thresholds for most groups. We therefore did not consider larger threshold shifts to avoid unsupported extrapolation. We also used the bootstrap-derived upper bound of each threshold from 1,000 bootstrap replicates as a higher-threshold case, which yields a lower-bound estimate of the exceedance proportion (i.e., higher thresholds imply lower exceedances). For each scenario, we recomputed the annual proportion of sites exceeding thresholds under SSP1-2.6, SSP2-4.5, and SSP5-8.5. These threshold shifts were not intended to represent empirically estimated acclimation magnitudes. Instead, they were used as bounding sensitivity scenarios to test how strongly potential increases in VPD thresholds could buffer projected exceedance risk across emissions pathways.

## Supplementary Material

Appendix 01 (PDF)

## Data Availability

All data used in this study are publicly available, including tree-ring width measurements (99), tree mortality records (6, 52, 100–103), monthly mean, maximum and minimum temperature, precipitation, actual vapor pressure, potential evapotranspiration (104), shortwave radiation (79) and the future scenario datasets in CMIP6 (82). Codes for the analysis are available from https://github.com/leefew0823-star/threshold.git.
