# Supplementary material for: Increasing risk of vapor pressure deficit exceeding critical thresholds for tree growth
Source: Proc Natl Acad Sci U S A. 2026 Sep 8;123(37):e2530363123. doi: 10.1073/pnas.2530363123 (PMC13578817; doi:10.1073/pnas.2530363123)
Supplement: Supplementary file 1 [file pnas.2530363123.sapp.pdf]

Supporting Information for

Increasing risk of vapor pressure deficit exceeding critical thresholds for tree growth

Tiewei Li[a], Hans W. Chen[b], Deliang Chen[a,c], Jingfeng Xiao[d], Lanlan Guo[e], Lianyou Liu[e], Feng Shi[f], Wenping Yuan[g], Ziqian Zhong[b], Huan Zheng[e], Bin He[a,1]

[a]Department of Earth System Science, Tsinghua University, Beijing, 100084, China.

[b]Department of Environmental and Energy Sciences, Chalmers University of Technology, SE-412 96 Gothenburg, Sweden.

[c]Institute for Carbon Neutrality, Tsinghua University, Beijing, 100084, China.

[d]Earth Systems Research Center, Institute for the Study of Earth, Oceans, and Space, University of New Hampshire, Durham, NH 03824, USA.

[e]Faculty of Geographical Science, Beijing Normal University, Beijing 100875, China.

[f]State Key Laboratory of Lithospheric and Environmental Coevolution, Institute of Geology and Geophysics, Chinese Academy of Sciences, Beijing 100029, China.

[g]Institute of Carbon Neutrality, Sino-French Institute for Earth System Science, College of Urban and Environmental Sciences, Peking University, Beijing 100871, China.

[1]To whom correspondence may be addressed. Email: *heb@tsinghua.edu.cn.*

Contents in this file include:

Supplementary Figures 1-18

Supplementary Tables 1-4

Supplementary Text 1

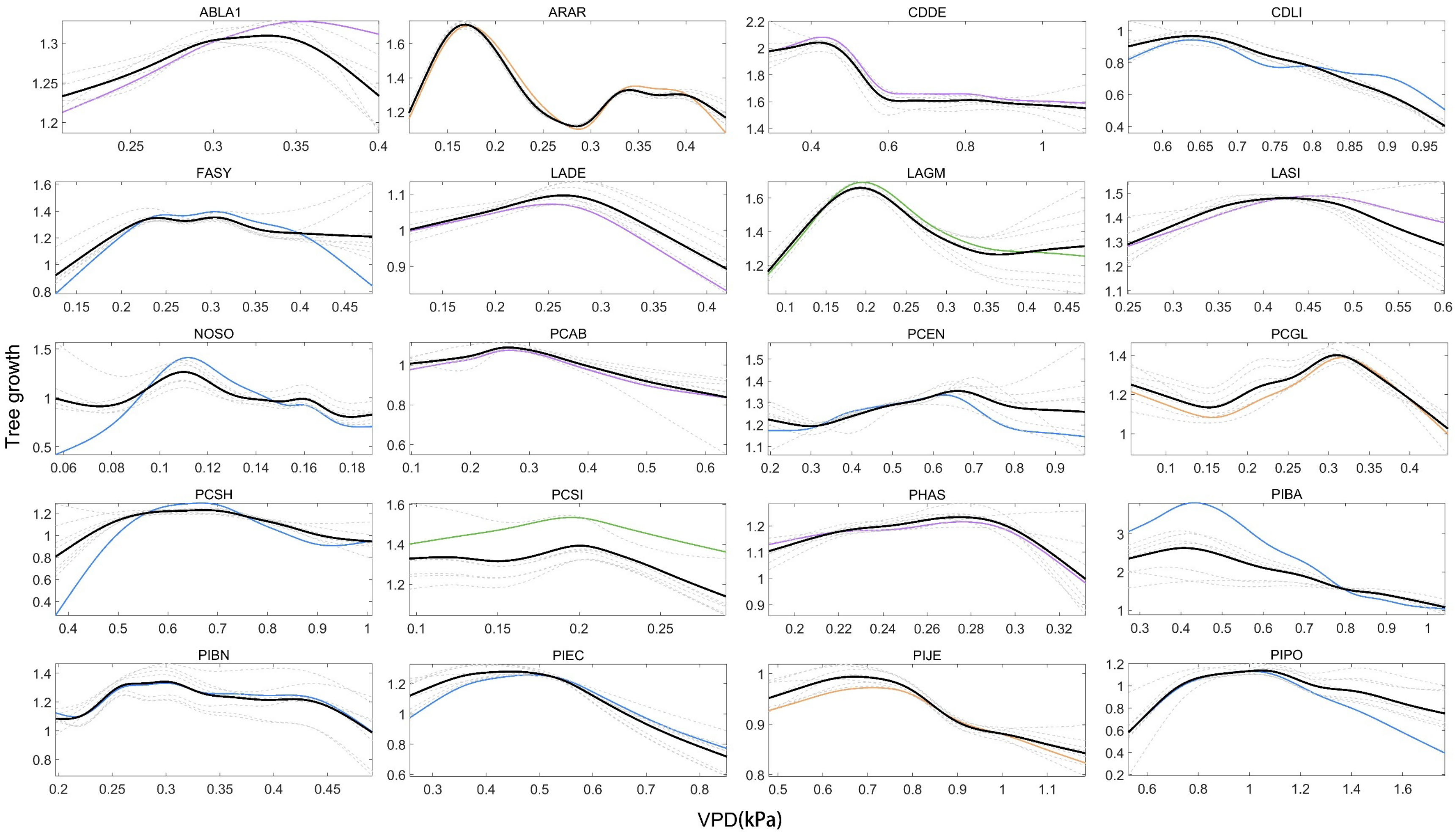

Fig. S1: The response of tree growth for each species group. The colored lines represent the curves that best fit the model, while the black lines represent the average curve of multiple models. The light gray dotted lines represent the fitted curves of other models.

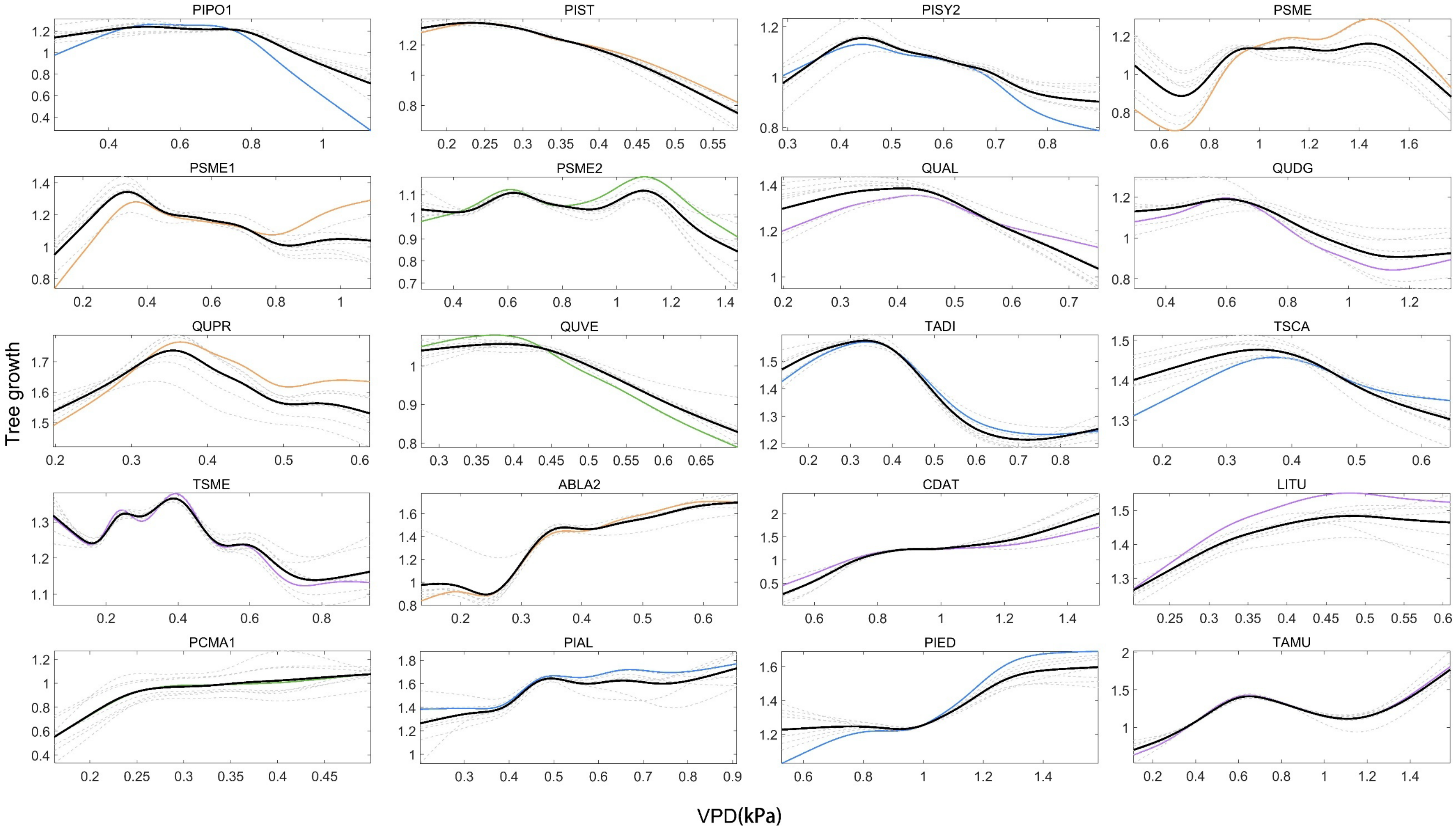

Fig. S1: Continued.

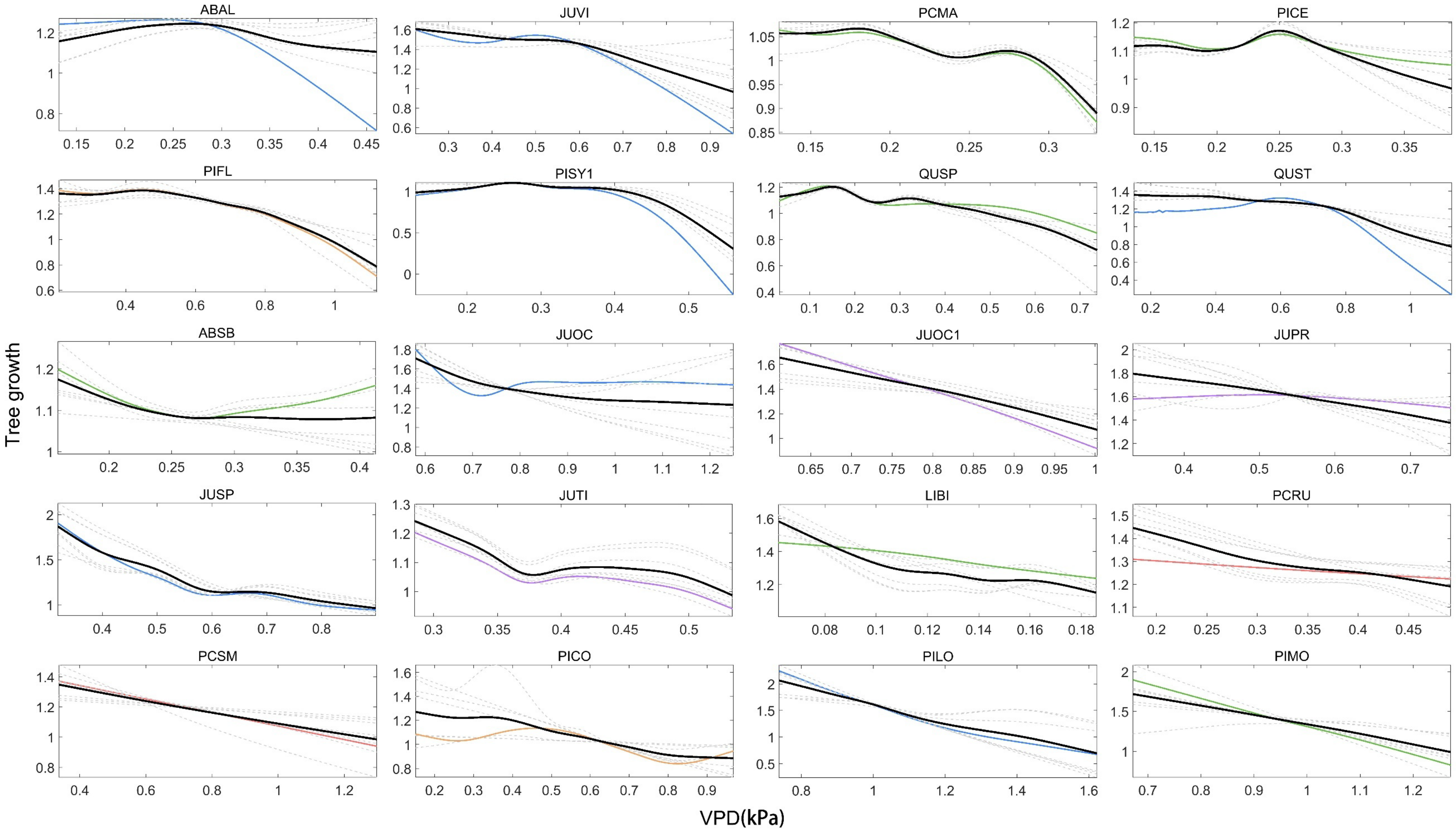

Fig. S1: Continued

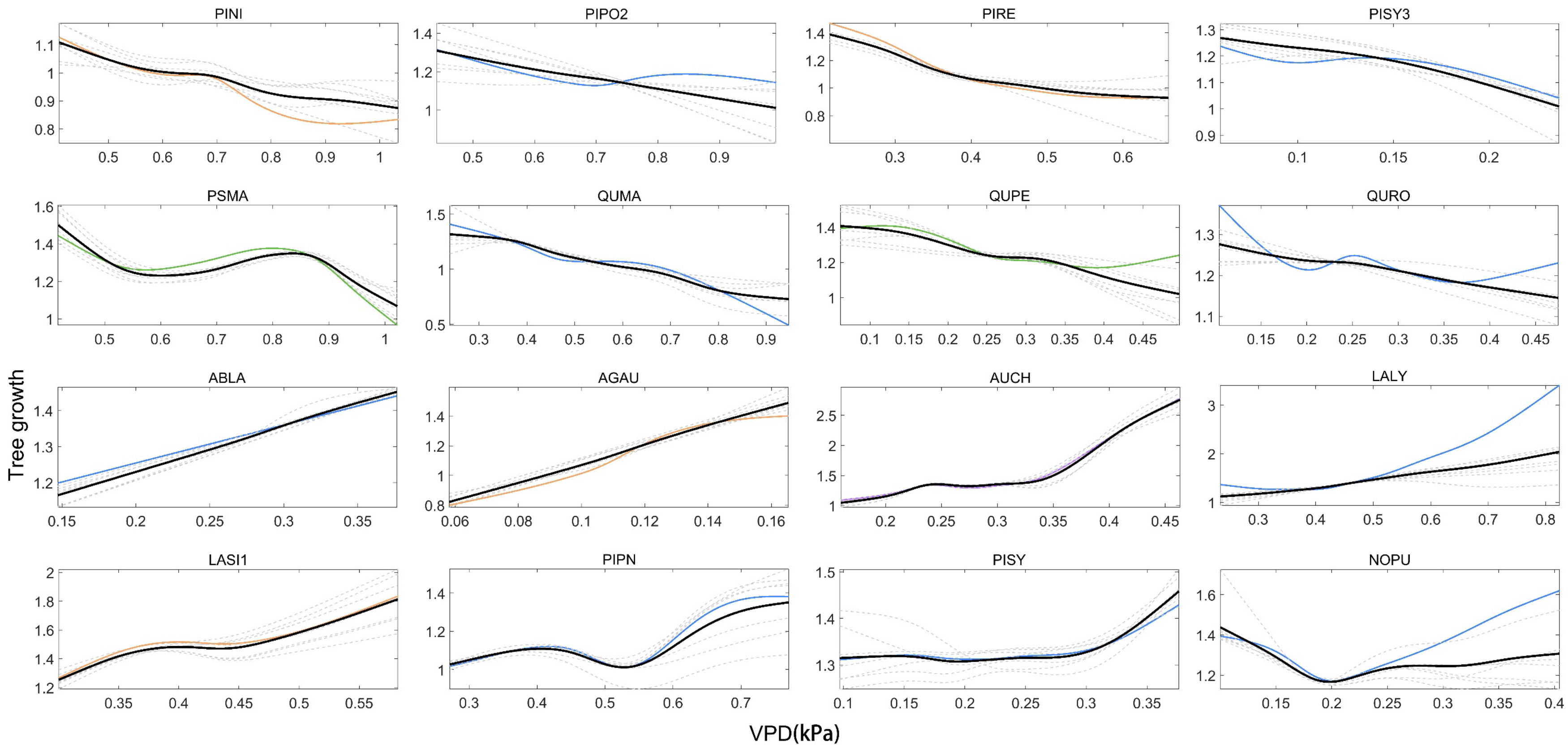

Fig. S1: Continued.

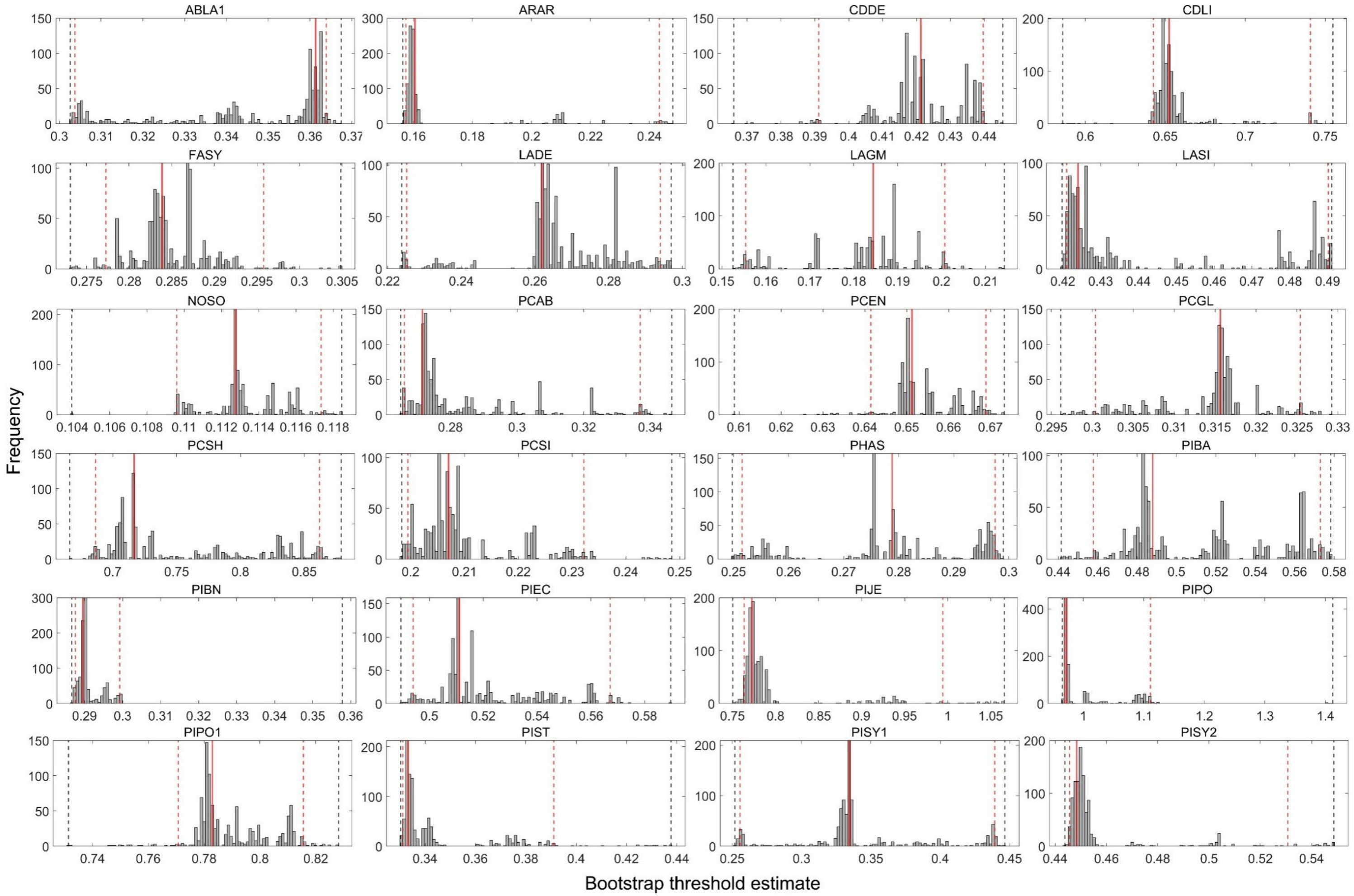

Fig. S2: Bootstrap threshold estimates for three types of tree growth responses to VPD (T1-T3). Gray bars show the distribution of threshold estimates from 1,000 bootstrap replicates. The gray column represents the histogram of the threshold estimation, the red vertical line indicates the estimated threshold, the red vertical dashed lines show the 95% confidence interval of the threshold estimation, and the black dash lines represent the maximum and minimum values of the threshold estimation.

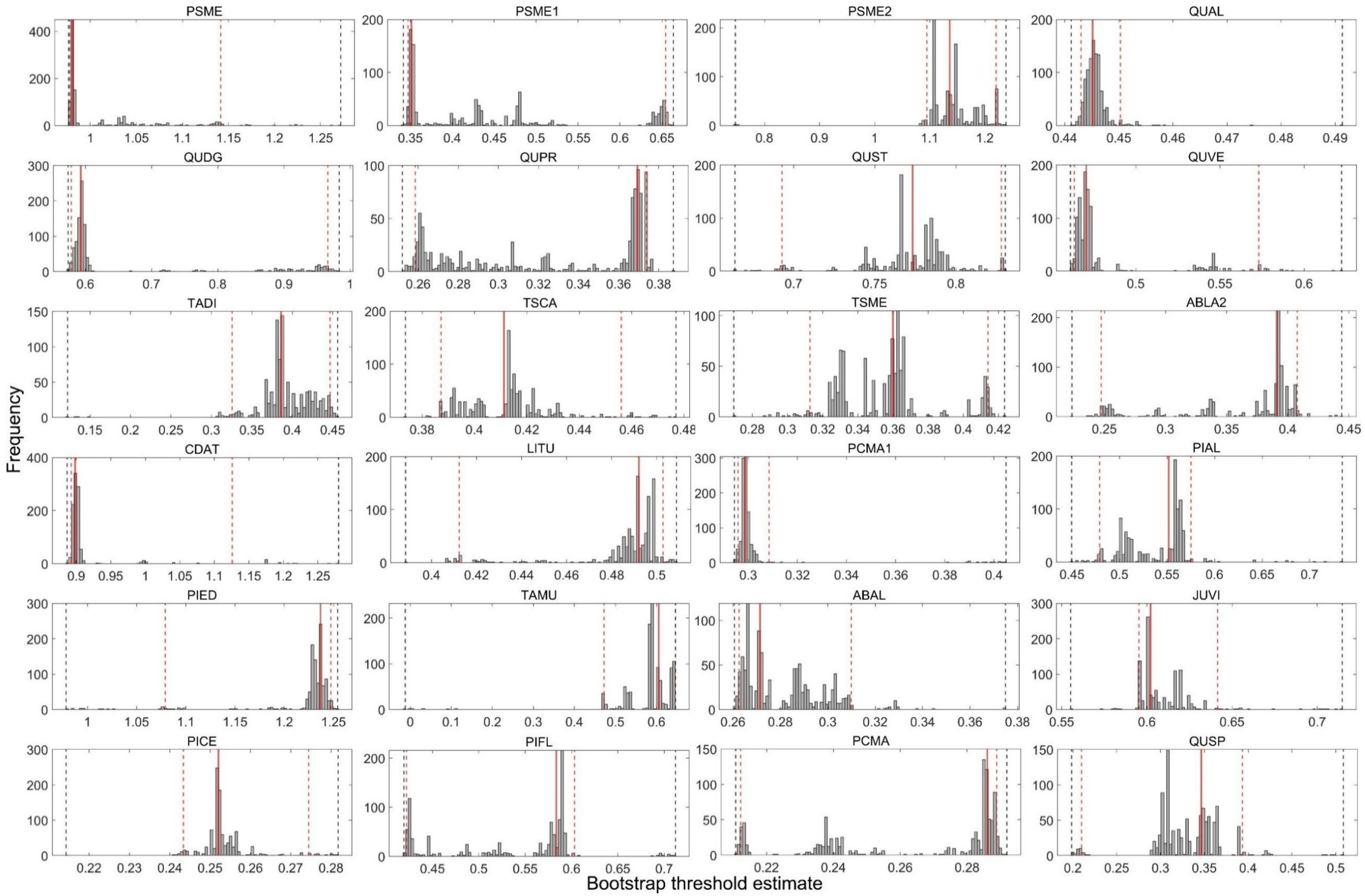

Fig. S2: Continued

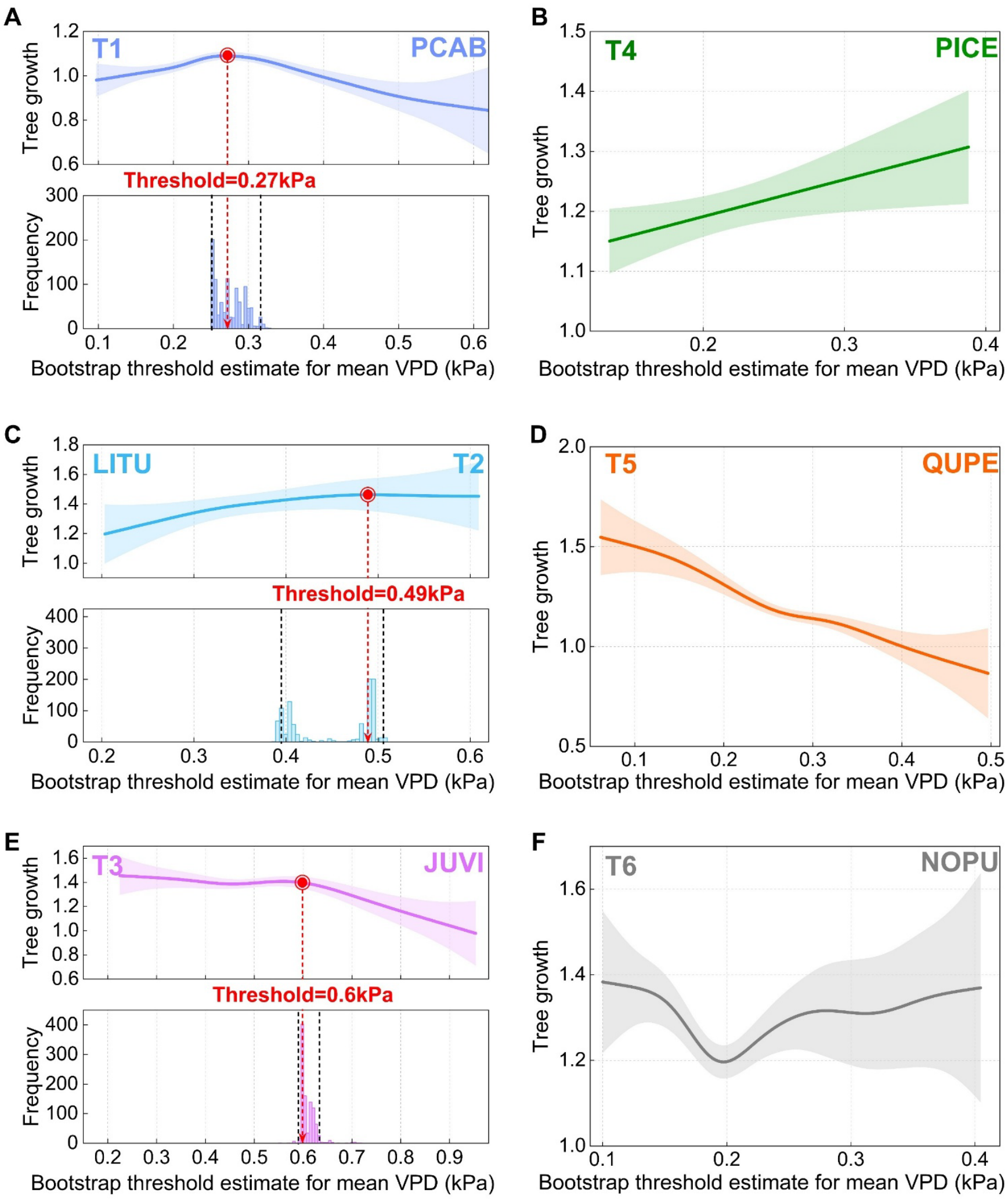

Fig. S3: Examples of the six types of tree growth responses to VPD: T1 (A), T2 (C), T3 (E), T4 (B), T5 (D), and T6 (F). The line graphs illustrate the marginal effects of VPD on tree growth obtained from the GAMs, and the bar graphs show the bootstrap threshold estimate for the mean VPD obtained from the TRMs. Shaded areas are 95% confidence intervals. The red dashed vertical line is the median threshold value and the black dashed vertical lines represent the 95% confidence interval.

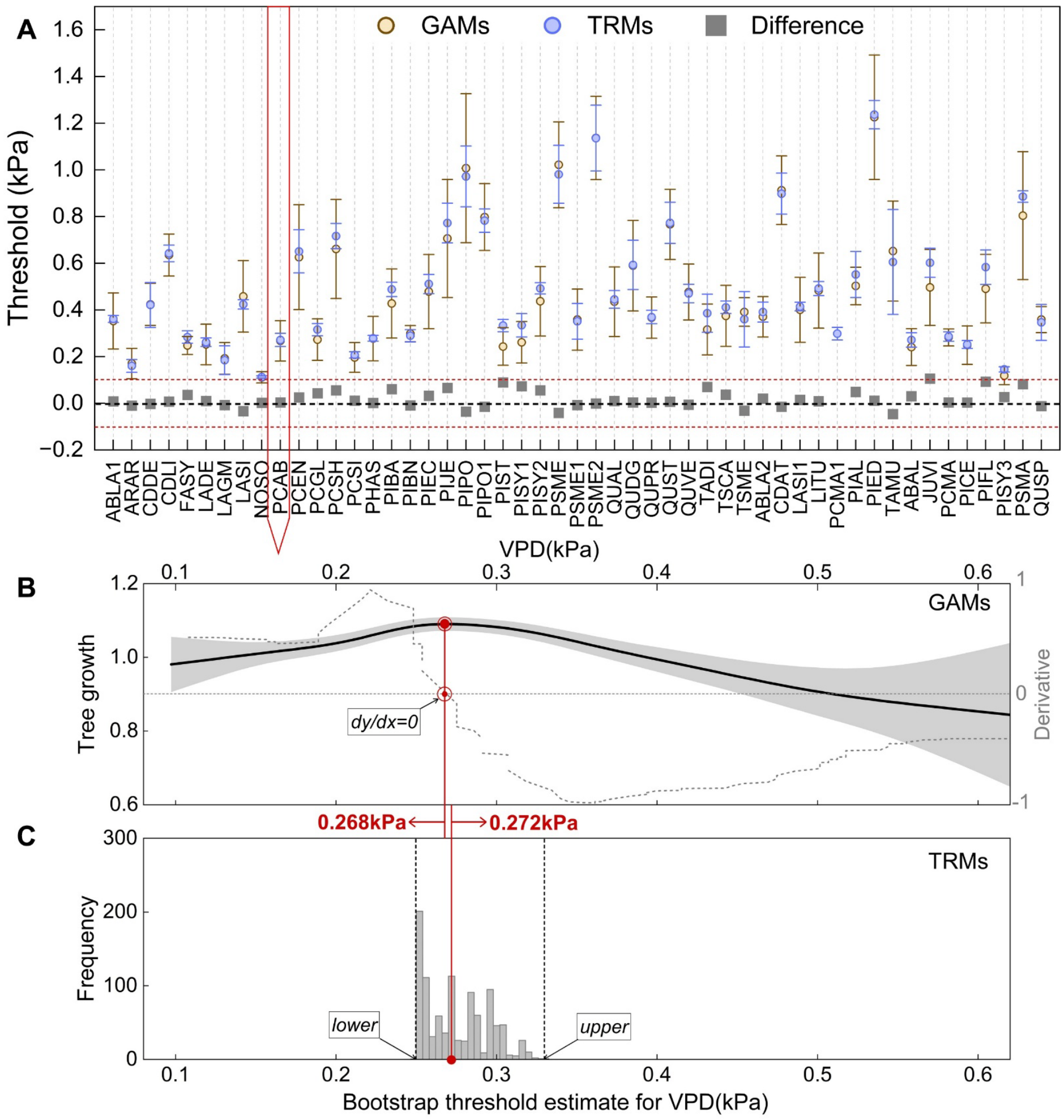

Fig. S4: Comparison of VPD threshold estimates from TRMs and GAMs. (A) Estimated thresholds from GAMs and TRMs and their differences. Brown points indicate thresholds identified as the zero-crossing of the first derivative of the GAMs marginal-effect curve. Error bars show uncertainty from 1,000 bootstrap resamples. Blue points indicate thresholds estimated from threshold regression models (TRMs). Error bars for both types of threshold estimates show uncertainty from 1,000 bootstrap resamples. Dark-gray boxes denote the differences between GAM- and TRM-based threshold estimates. The red dashed lines mark ±0.1 kPa. **(B, C)** Example of GAM-based and TRM-based derivation of threshold for the species group PCAB. The line graph illustrates the marginal effects of VPD on tree growth obtained from GAMs. Shaded area are 95% confidence intervals. In (B), the gray dashed line illustrates the first derivative of the GAM-estimated marginal effect. In (C) The bar graphs show the bootstrap threshold estimate for mean VPD obtained from TRMs. Black dashed vertical lines represent lower and upper bound of threshold estimate.

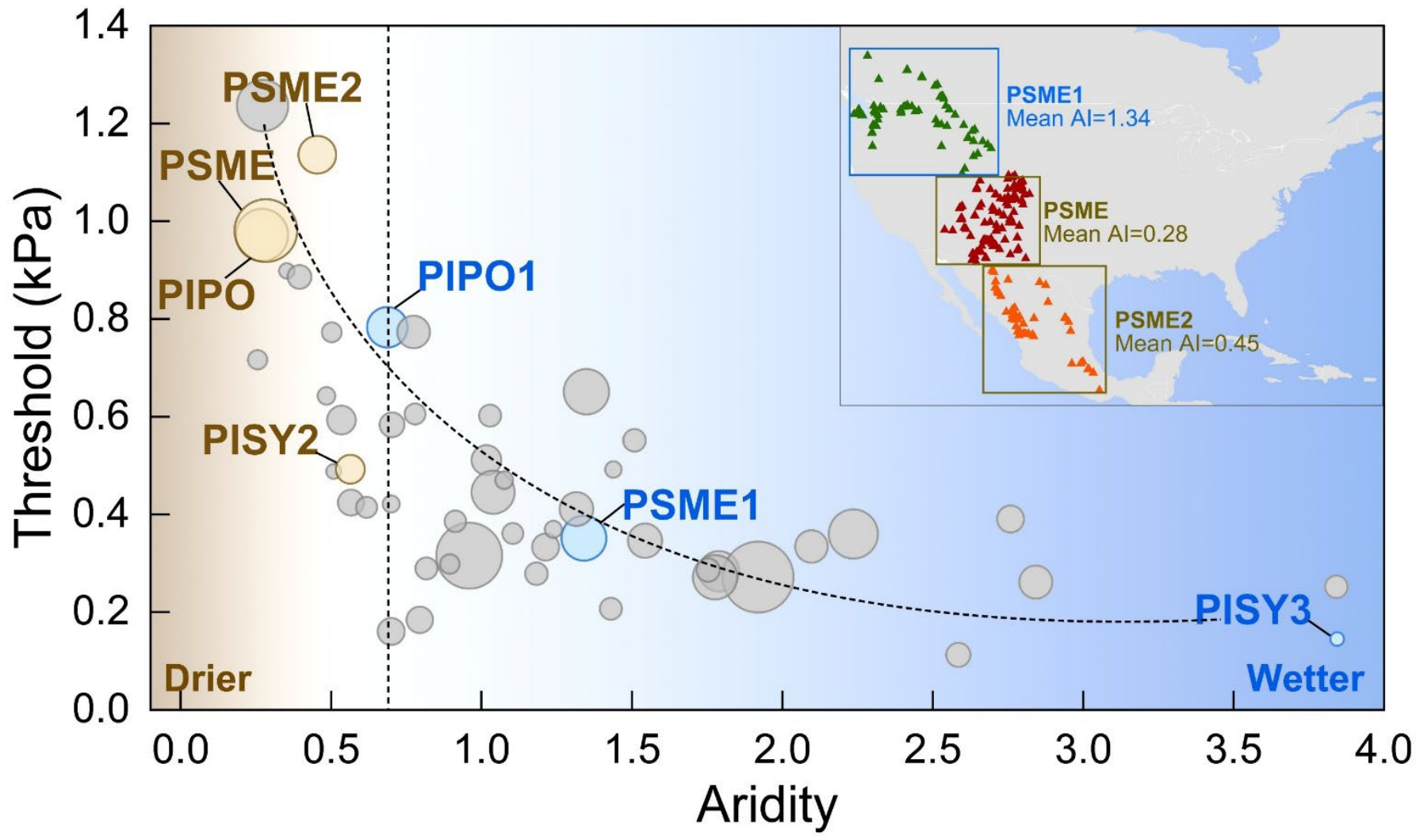

Fig. S5: Relationship between species-specific VPD thresholds and the aridity index (AI). Point size denotes the sample size for each species group. The vertical black dashed line marks AI = 0.65, which was used to separate arid and humid regions; the brown-to-blue background gradient indicates the transition from arid to humid conditions. Labeled species codes identify the three widespread species that occur under contrasting aridity conditions: *Pseudotsuga menziesii* (PSME), *Pinus ponderosa* (PIPO), *and Pinus sylvestris* (PISY). Brown points indicate species distributed in arid regions (PSME2, PSME, PIPO, and PISY2), blue points indicate species distributed in humid regions (PIPO1, PSME1, and PISY3), and gray points indicate the remaining species groups. The inset map shows *Pseudotsuga menziesii* as an example, with rectangular boxes used to visually distinguish the site clusters assigned to different species groups under contrasting geographic and aridity conditions.

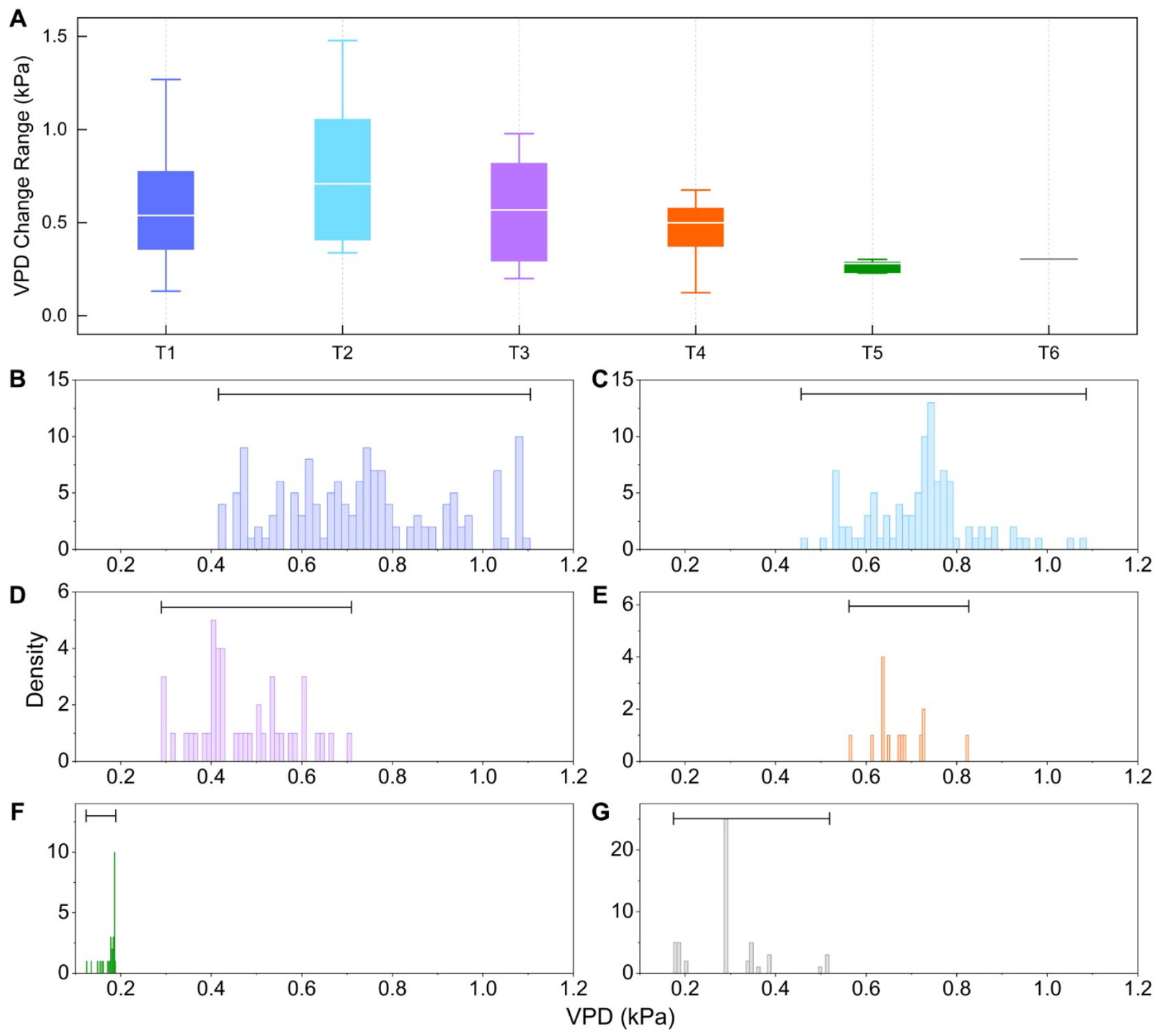

Fig. S6: Changes in species density along the VPD gradient. (A) Distribution of the historical

observed growing-season VPD range during 1901-2012 for each VPD–growth relationship type (T1–T6), shown as boxplots. **(B–G)** Species density across historical observed VPD bins along the VPD gradient for T1–T6.

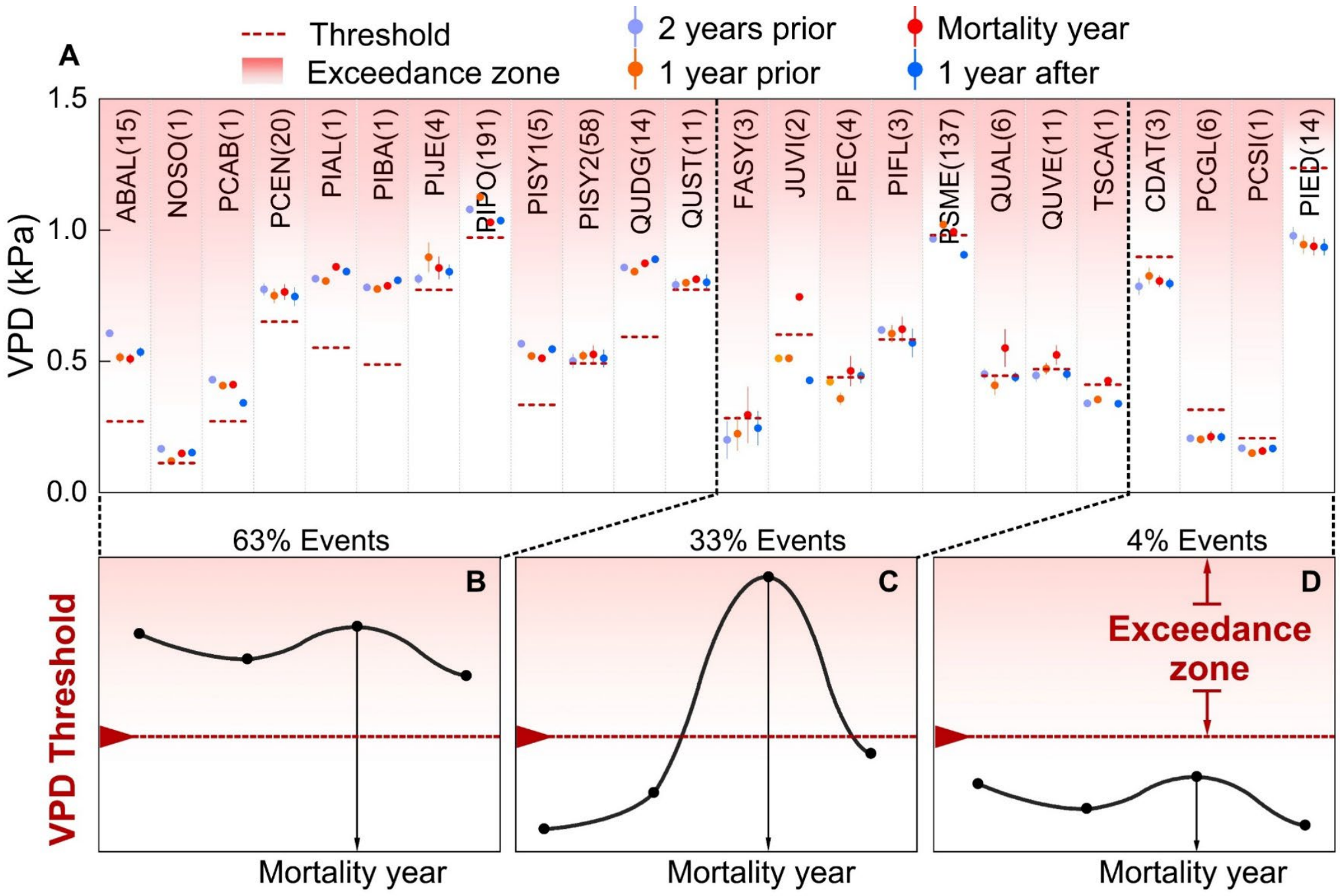

Fig. S7: Association between VPD threshold exceedance and tree mortality events. (A) VPD during the four-year mortality window, two years before the recorded mortality year, the mortality year, and the following year, shown relative to the VPD threshold (red dashed line). Points represent VPD values for matched mortality events in each year of the window, and error bars indicate the standard error of the mean. The red gradient region denotes the VPD exceedance zone (VPD above the threshold) for visualization. **(B–D)** Conceptual schematics illustrating the two threshold-exceedance patterns identified: multi-year exceedance and exceedance in the mortality year across the four-year mortality window. The solid black line represents the VPD change, and the red dashed line represents the VPD threshold. About 62.8% showed VPD above the corresponding threshold throughout the four-year mortality window, representing 12 species (A, B); 32.6% showed threshold exceedance in the mortality year, representing eight species (A, C), and 4.0% showed no threshold exceedance during the mortality window (A, D).

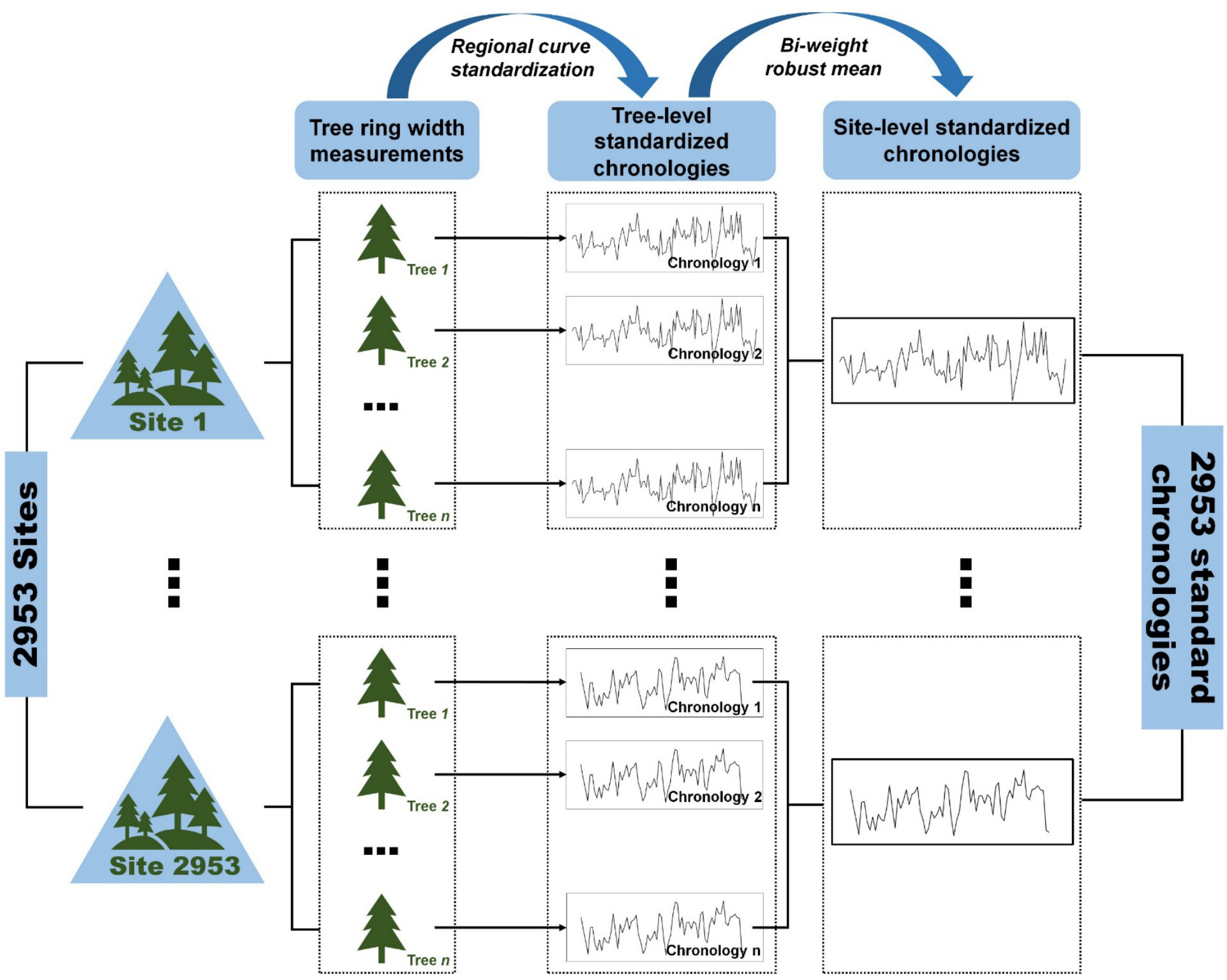

Fig. S8: Flow chart for processing tree ring data.

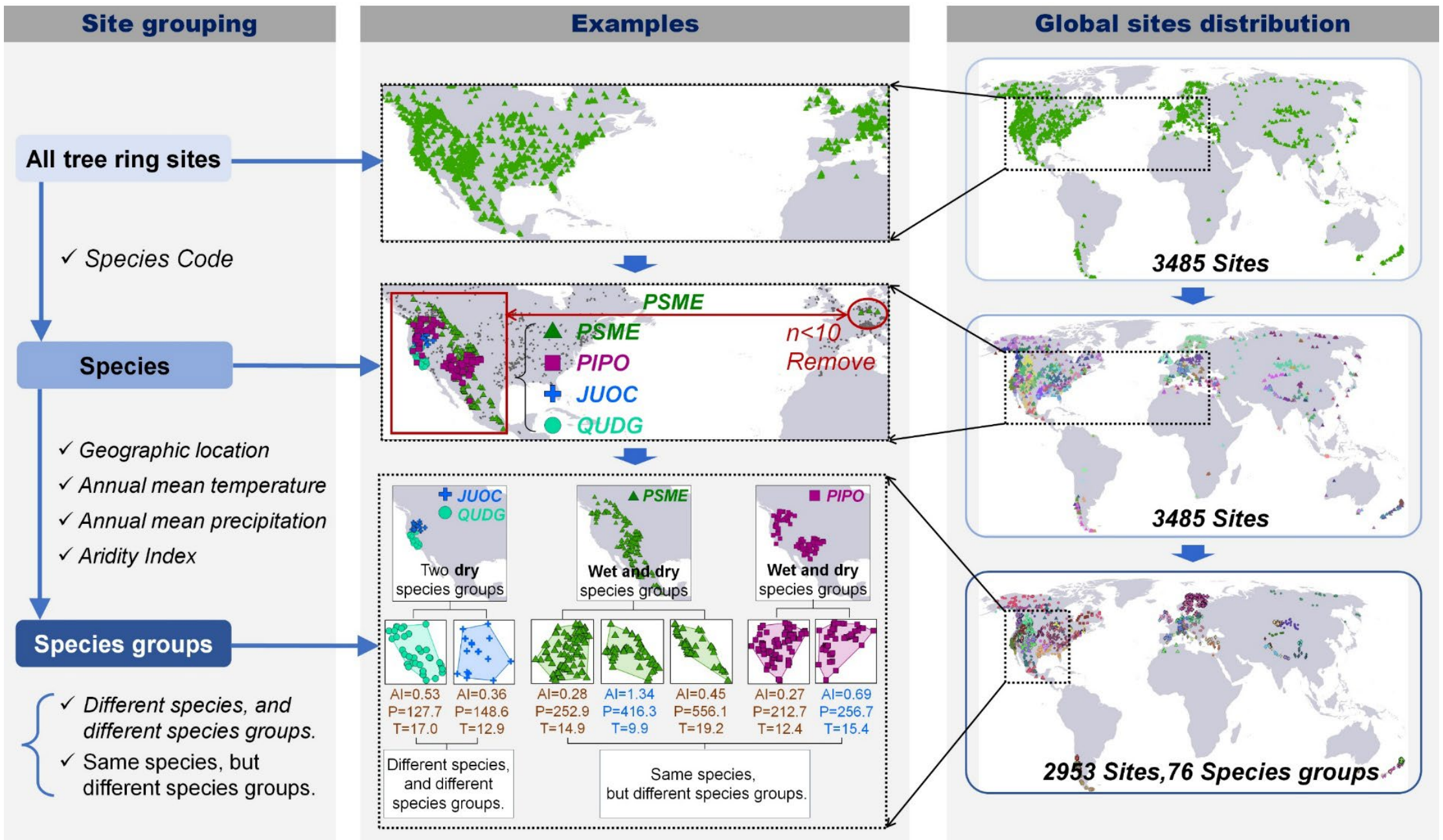

Fig. S9: Schematic of tree-ring site grouping. Tree-ring sites were first grouped by species code. For widely distributed species, sites were further separated into species groups according to geographic location and baseline climate conditions, including mean annual temperature, mean annual precipitation, and aridity index. Geographically isolated sites were assigned to separate groups when the sample size was sufficient ($n \geq 10$); otherwise, they were excluded from subsequent threshold analyses. The example shows that the same species can be separated into different species groups when it occurs under contrasting geographic and aridity conditions. The global maps show the distribution of all tree-ring sites before grouping, after species-level grouping, and after filtering

species groups with fewer than 10 sites. Rectangles and dashed boxes are used as visual guides to illustrate the grouping workflow.

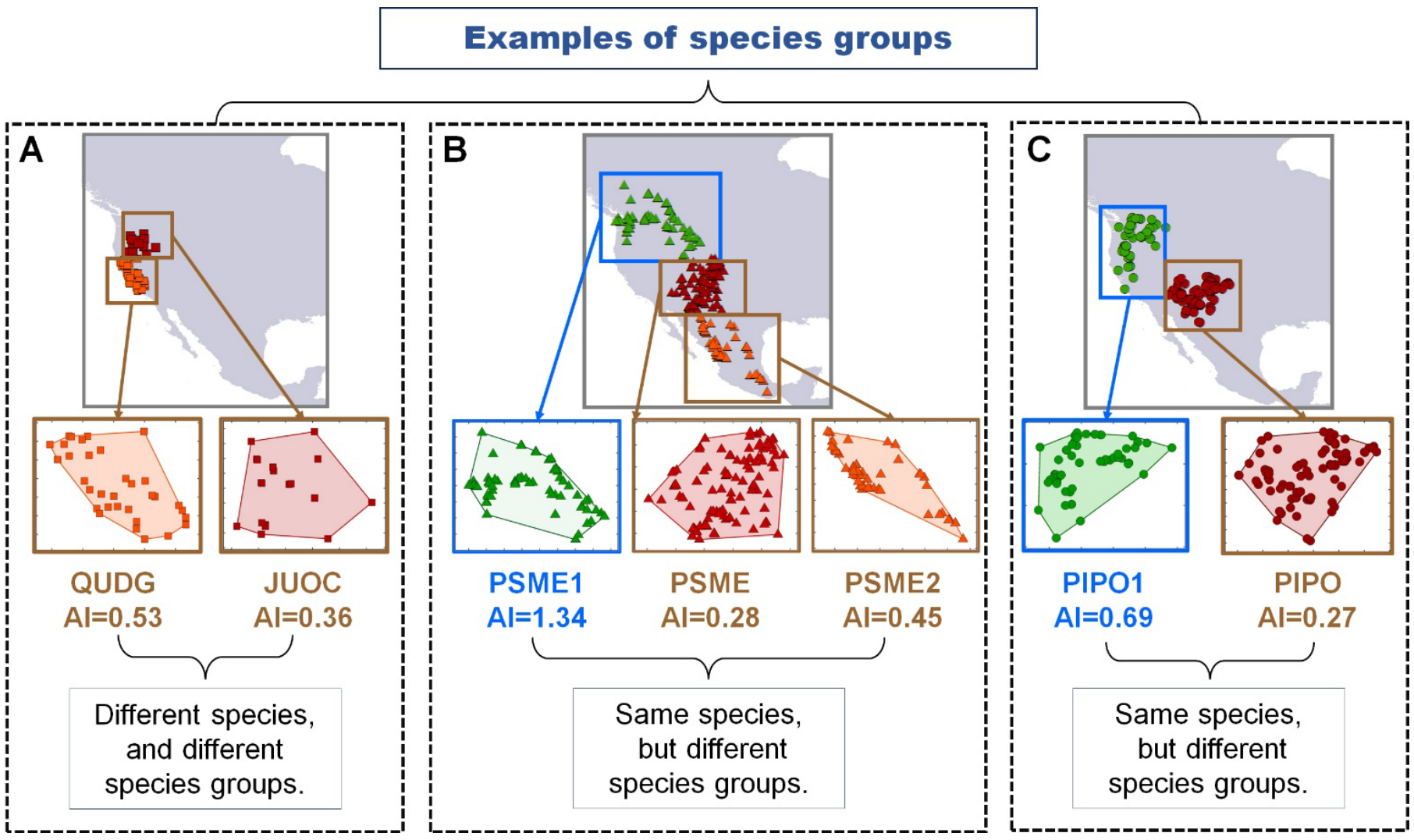

Fig. S10: Examples illustrating the definition of species groups. A species group refers to a subset of tree-ring sites from the same species that occur under comparable geographic and baseline climate conditions. (A) Different species are treated as different species groups. (B, C) For widely distributed species, sites from the same species can be separated into different species groups when they occur under contrasting geographic and aridity conditions. For example, *Pseudotsuga menziesii* was separated into PSME1, PSME, and PSME2, and *Pinus ponderosa* was separated into PIPO1 and PIPO. AI denotes the aridity index, with lower values indicating drier conditions. Colored points represent tree-ring sites within each species group, and the corresponding colored polygons represent convex hulls constructed from the sites of each species group. These convex hulls are used to illustrate the geographic extent represented by the tree-ring sites and do not represent taxonomic boundaries or fixed ecological zones.

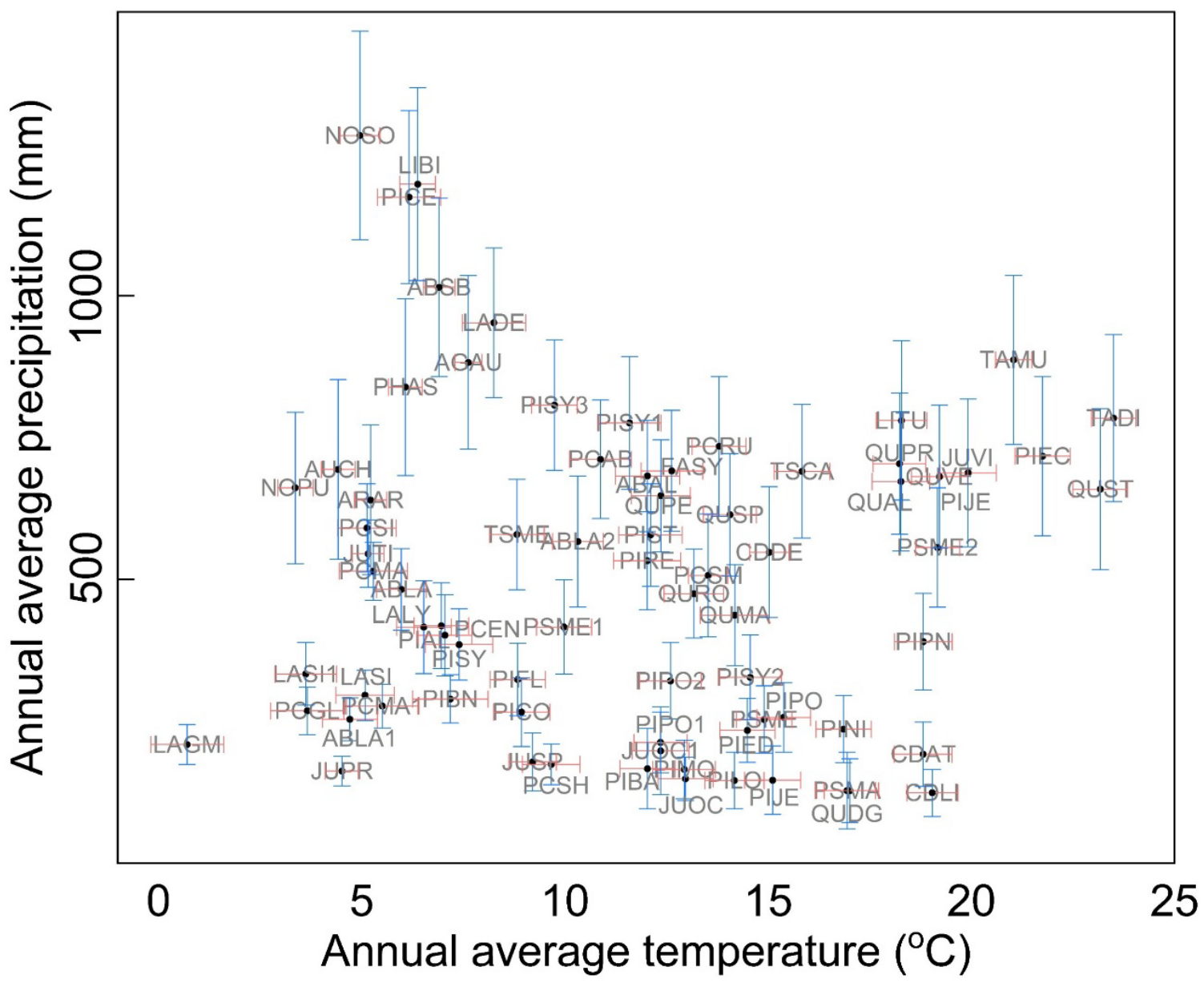

Fig. S11: The average temperature and precipitation and corresponding standard deviations of the 76 species groups.

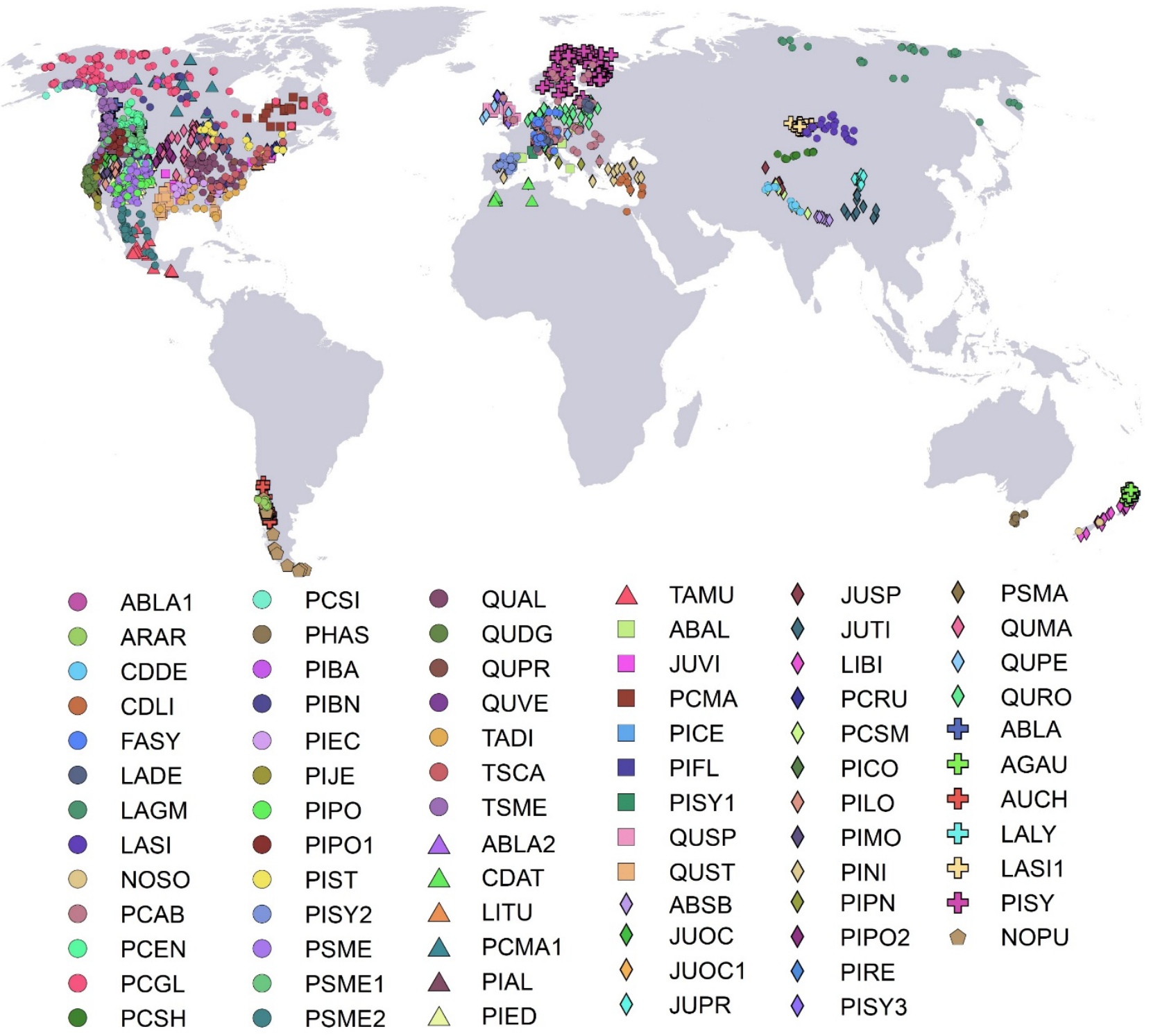

Fig. S12: The spatial distribution of each tree ring site obtained from the International Tree Ring Data Bank (ITRDB). Circle, triangle, square, diamond, cross, and pentagon markers represent response types T1–T6, respectively; colors denote different tree species groups.

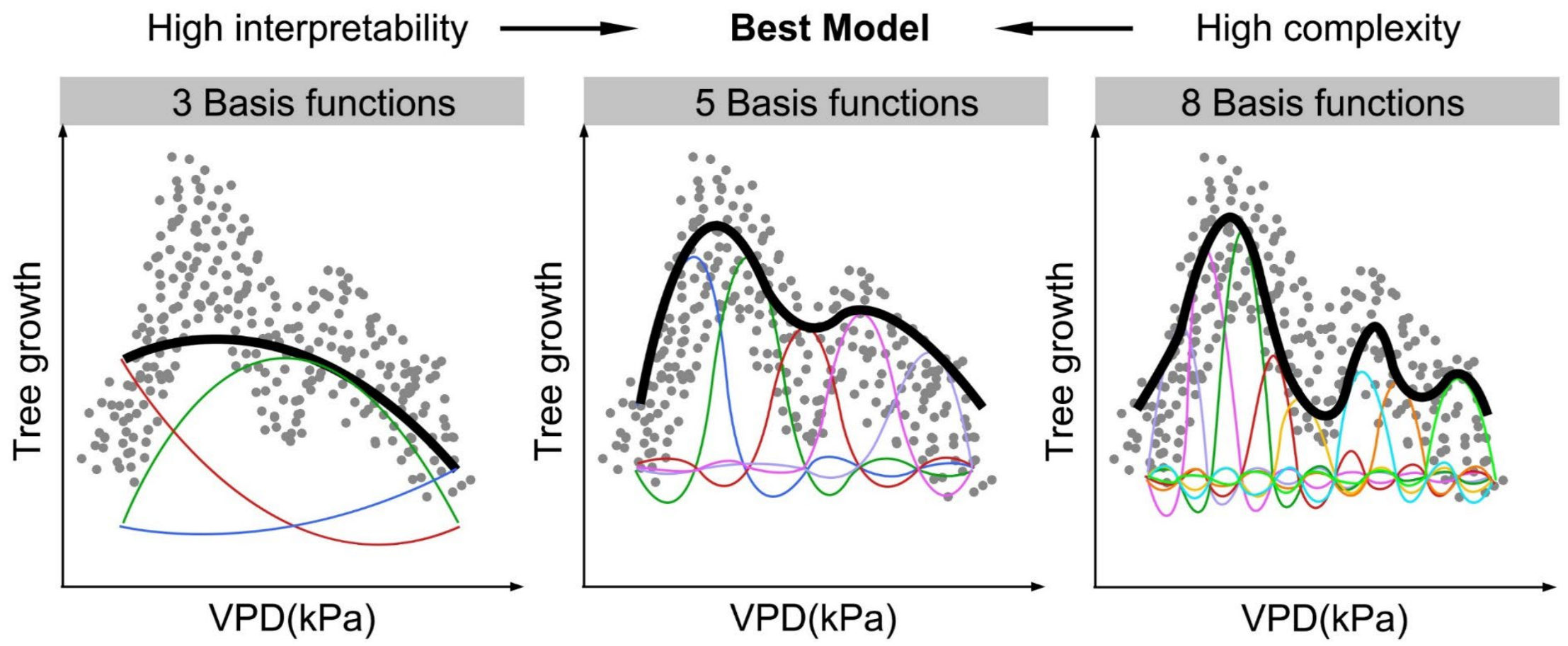

Fig. S13: Conceptual representation of a generalized additive model. Colored lines represent different basis functions. Black solid lines represent the response curves of tree growth to VPD obtained from multiple basis functions. The model incorporating three basis functions has higher interpretability, but it cannot reflect the change of the data. The model incorporating eight basis functions is too complex to analyze trends of the data. Therefore, the model incorporating five basis functions achieves a trade-off between high interpretability and high complexity and is considered the best model.

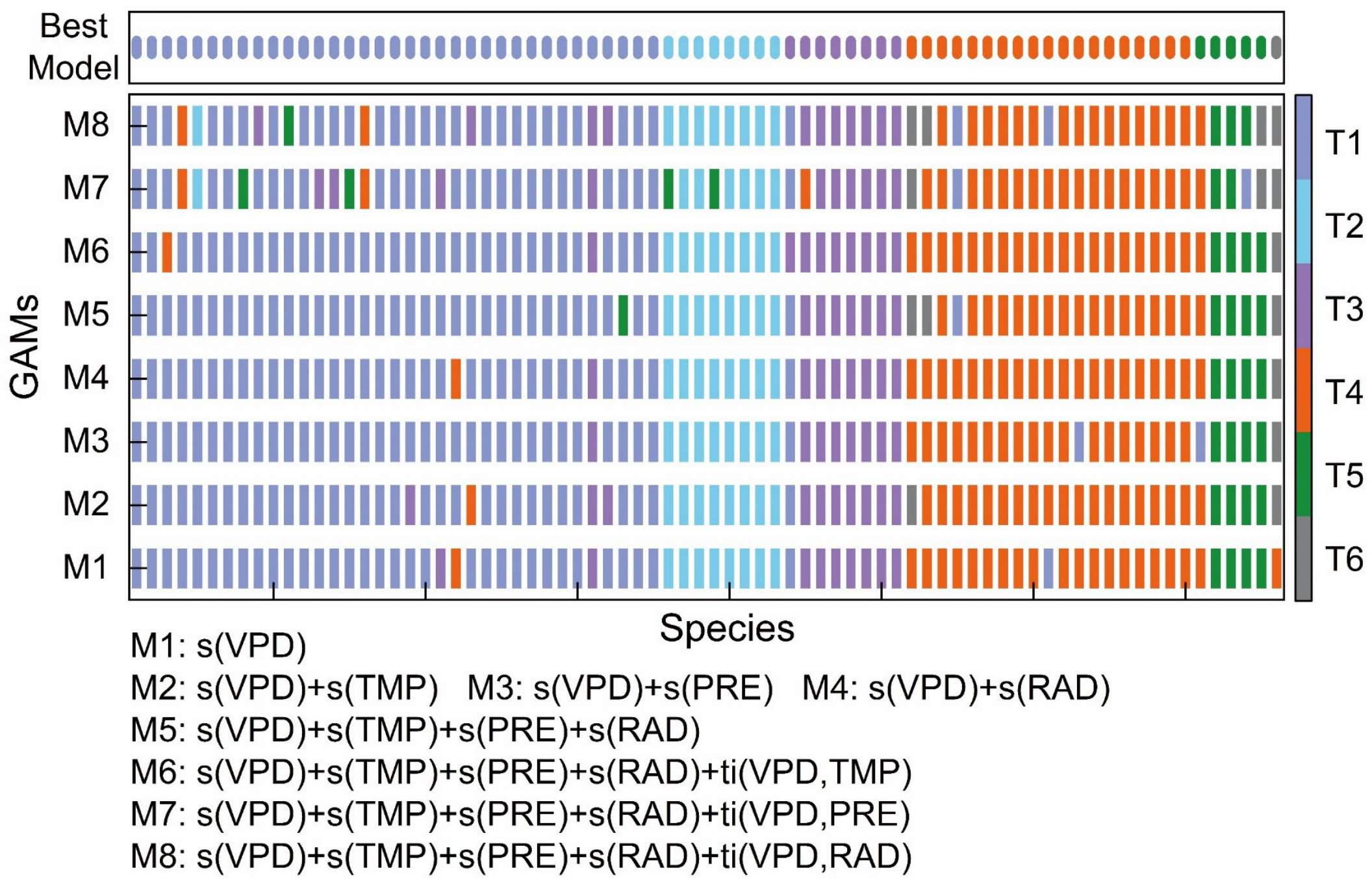

Fig S14: Consistency of the nonlinear VPD–growth relationships fitted by different GAM models.

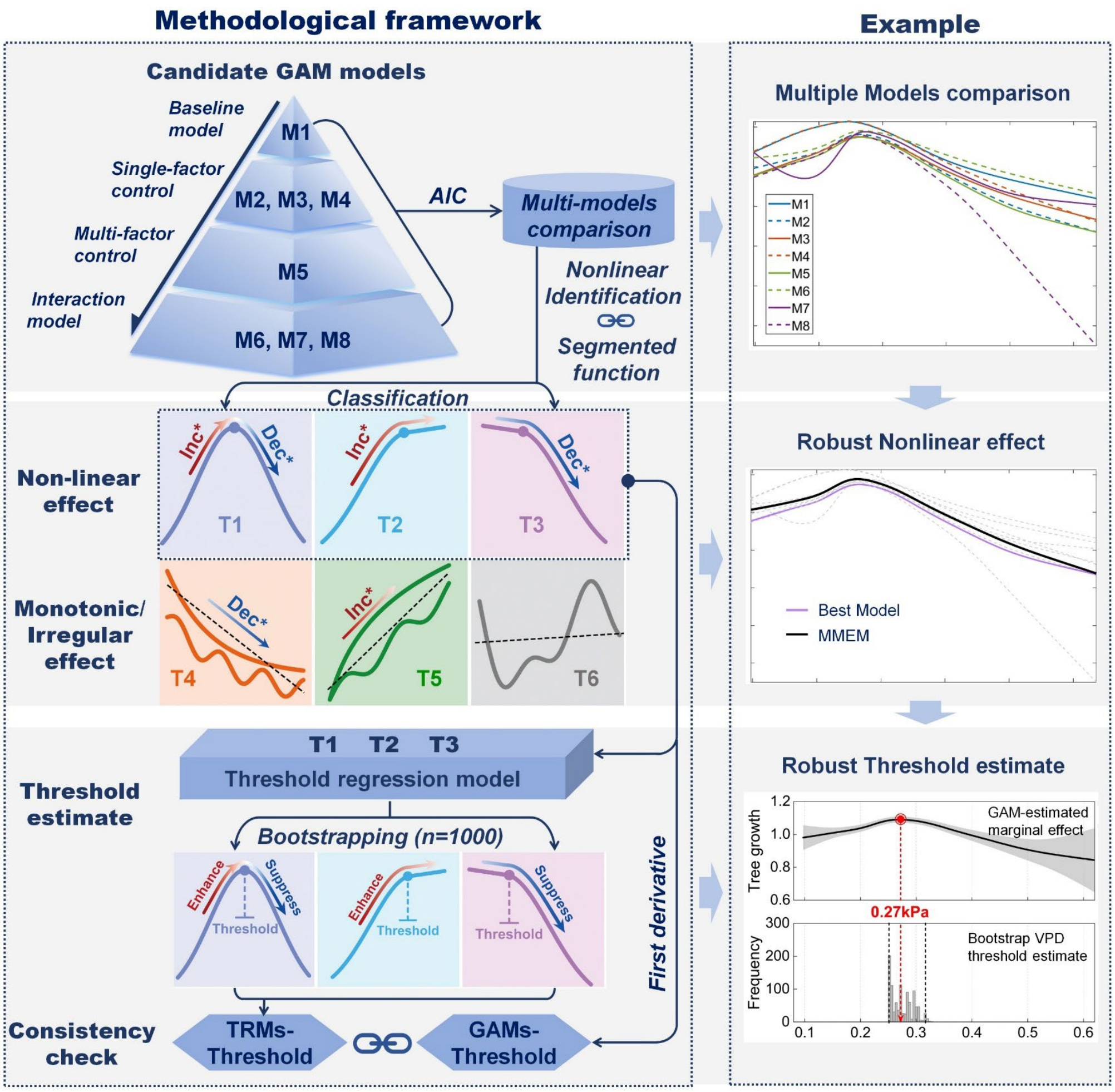

Fig. S15: Methodological framework used in this study.

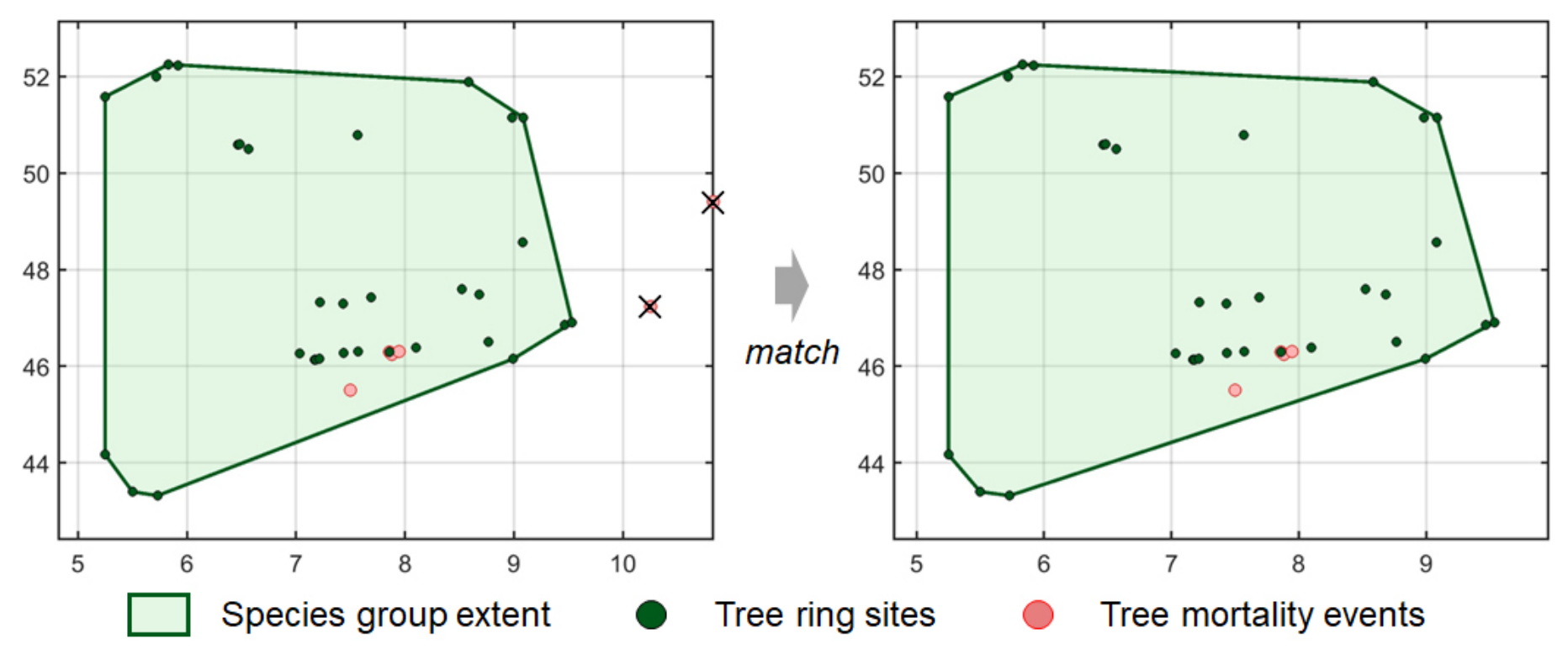

Fig. S16: Schematic of matching tree mortality records with species groups. The geographic extent of a species group was defined as the convex hull constructed from all tree-ring sites within that species group. Tree mortality records were matched to a species group only when they had the same species code and their locations fell within the convex hull of that species group. Green points represent tree-ring sites, the light-green polygon represents the convex hull of the species group, red points represent matched tree mortality records, and black crosses represent mortality records outside the species group extent that were not matched.

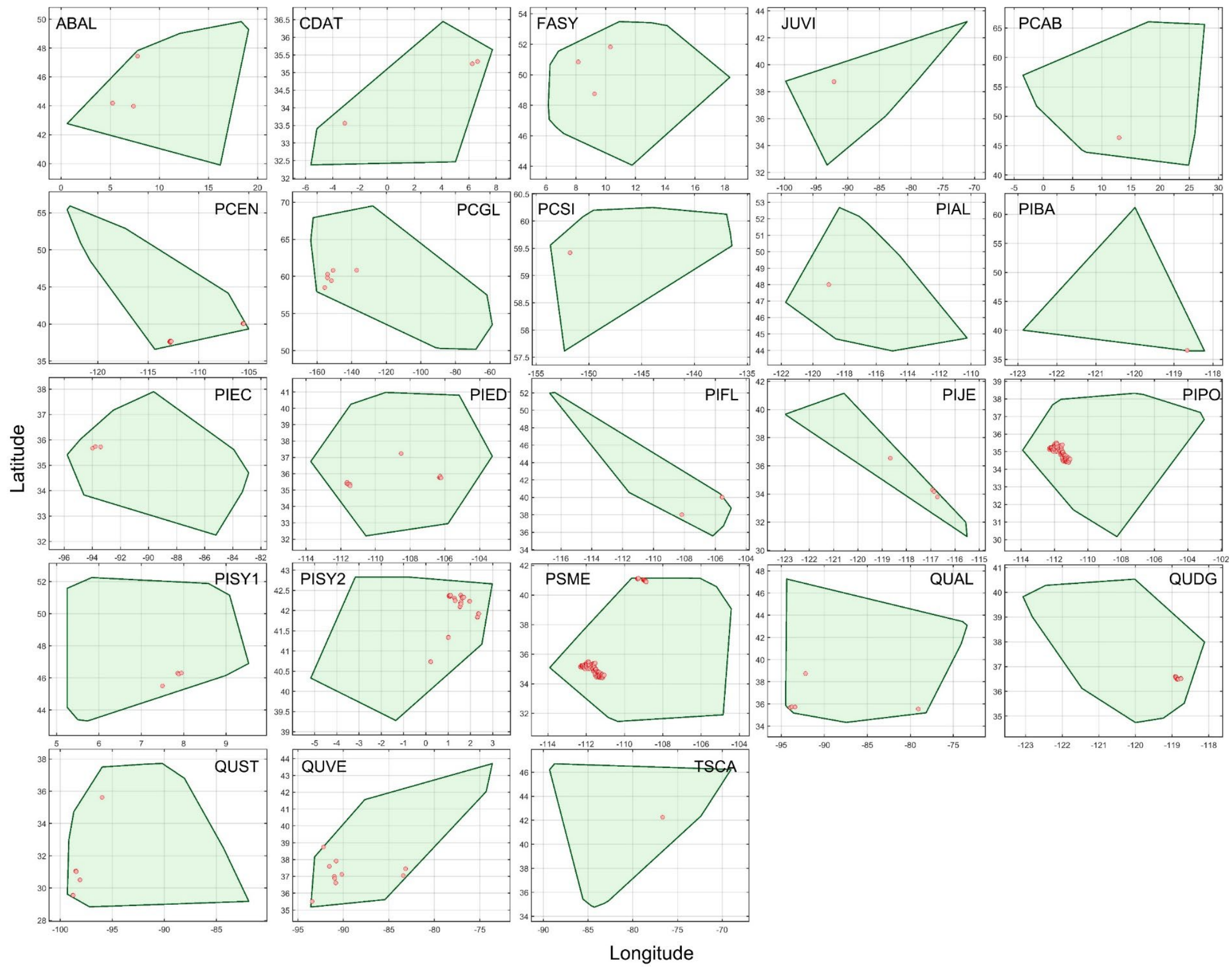

Fig. S17: Matched tree mortality records within the geographic extent of each species group. Each panel shows one species group. The light-green polygon represents the convex hull constructed from all tree-ring sites within that species group, green points represent tree-ring sites, and red points represent matched tree mortality records. Mortality records were matched only when they had the same species code and occurred within the convex hull of the corresponding species

group. For PIPO and PSME, multiple mortality records were documented at the same locations, resulting in overlapping red points.

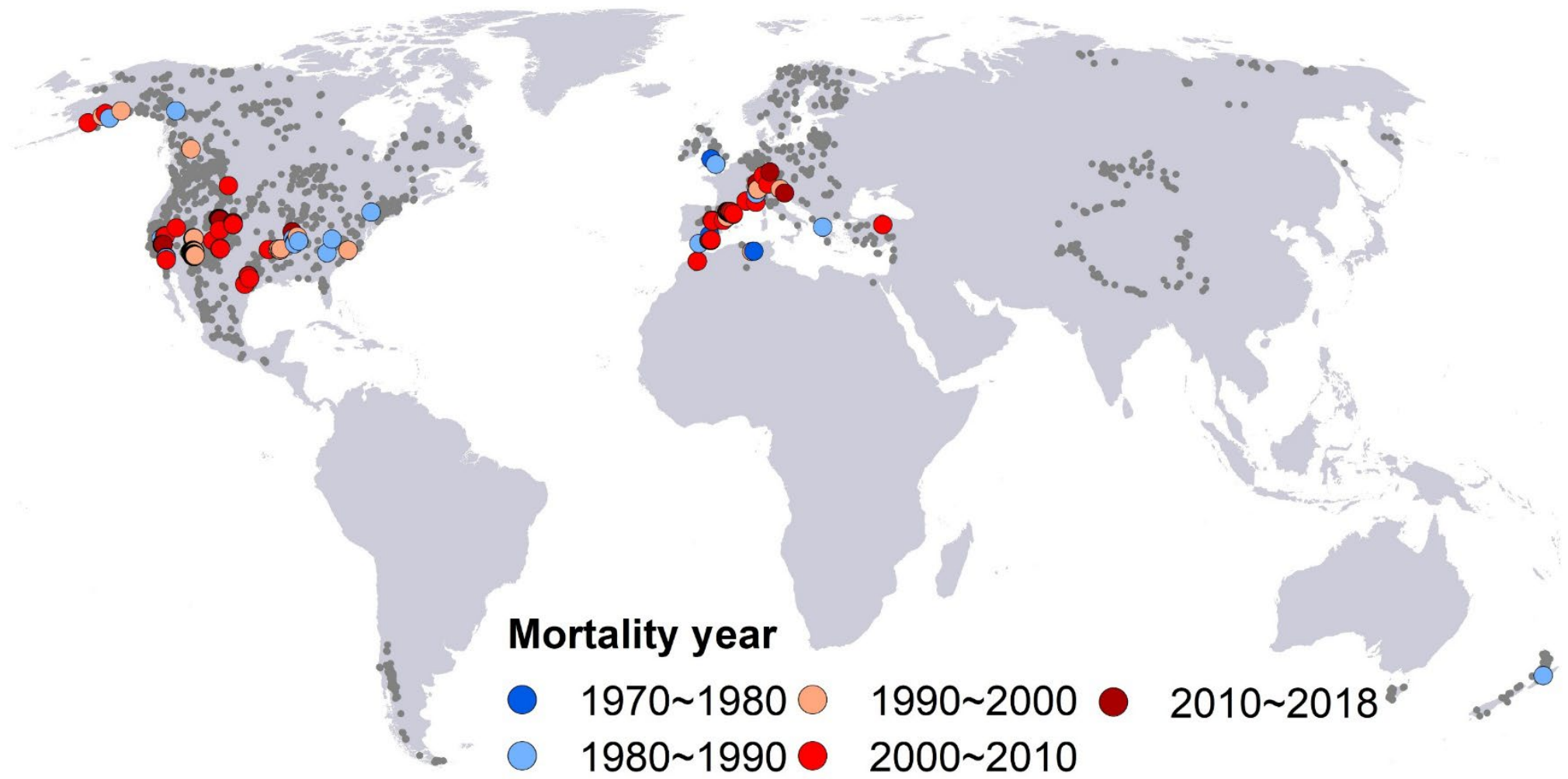

Fig. S18: Global distribution of tree mortality events during 1970–2018. Circles are color coded according to the year of mortality and gray dots represent tree ring sites.

Table S1: Species group code, response types, VPD threshold, mean aridity index (AI) of tree species, and the number of tree ring sites (*N*), *p-value* and *edf* for each tree species.

Species Code	Type	Threshold(kPa)	Mean AI	*N*	*p-value*	edf
ABLA1	T1	0.36	1.10	19	<0.01	3.217
ARAR	T1	0.16	0.70	30	<0.001	7.413
CDDE	T1	0.42	0.70	13	<0.001	6.449
CDLI	T1	0.65	0.48	14	<0.001	2.265
FASY	T1	0.28	1.79	64	<0.001	5.675
LADE	T1	0.26	2.84	44	<0.001	3.297
LAGM	T1	0.18	0.79	29	<0.001	5.276
LASI	T1	0.42	0.57	28	<0.1	2.705
NOSO	T1	0.11	2.58	25	<0.001	7.663
PCAB	T1	0.27	1.92	186	<0.001	5.350
PCEN	T1	0.65	1.35	79	<0.001	7.025
PCGL	T1	0.32	0.96	161	<0.001	7.099
PCSH	T1	0.72	0.26	16	<0.001	5.573
PCSI	T1	0.21	1.43	21	<0.01	4.046
PHAS	T1	0.28	1.18	22	<0.005	2.063
PIBA	T1	0.49	0.51	10	<0.001	4.013
PIBN	T1	0.29	0.82	21	<0.001	6.628
PIEC	T1	0.51	1.02	36	<0.001	4.143
PIJE	T1	0.77	0.50	17	<0.001	3.685
PIPO	T1	0.97	0.27	103	<0.001	7.294
PIPO1	T1	0.78	0.69	63	<0.001	6.346

Species Code	Type	Threshold(kPa)	Mean AI	*N*	*p-value*	edf
PIST	T1	0.33	1.21	30	<0.001	3.541
PISY2	T1	0.45	0.56	33	<0.001	5.533
PSME	T1	0.98	0.28	147	<0.001	8.069
PSME1	T1	0.35	1.34	77	<0.001	7.437
PSME2	T1	1.14	0.45	55	<0.001	7.40477
QUAL	T1	0.45	1.04	72	<0.001	4.795
QUDG	T1	0.59	0.53	33	<0.001	4.981
QUPR	T1	0.37	1.24	13	<0.001	5.120
QUVE	T1	0.47	1.08	14	<0.01	2.612
TADI	T1	0.39	0.91	20	<0.001	5.342
TSCA	T1	0.41	1.32	45	<0.1	2.678
TSME	T1	0.36	2.24	93	<0.001	8.275
ABLA2	T2	0.39	2.76	30	<0.001	8.089
CDAT	T2	0.90	0.35	11	<0.001	5.541
LITU	T2	0.49	1.44	12	<0.1	2.288
PCMA1	T2	0.30	0.89	16	<0.05	2.362
PIAL	T2	0.55	1.51	22	<0.001	6.881
PIED	T2	1.24	0.27	101	<0.001	4.691
TAMU	T2	0.61	0.78	19	<0.001	5.977
ABAL	T3	0.27	1.78	75	<0.001	2.998
JUVI	T3	0.60	1.03	21	<0.001	4.268
PCMA	T3	0.29	1.75	23	<0.001	5.027
PICE	T3	0.25	3.84	22	<0.05	2.317
PIFL	T3	0.58	0.70	26	<0.1	2.024
PISY1	T3	0.33	2.10	42	<0.1	4.826
QUSP	T3	0.35	1.54	47	<0.001	7.121
QUST	T3	0.77	0.77	43	<0.001	5.072
ABSB	T4		1.44	11	0.7	1.000
JUOC	T4		0.36	23	<0.001	4.273
JUOC1	T4		0.67	10	<0.001	1.691
JUPR	T4		0.21	16	<0.1	2.313
JUSP	T4		0.39	19	<0.001	6.955
JUTI	T4		0.73	12	0.17	3.680
LIBI	T4		2.85	25	<0.001	4.574
PCRU	T4		1.64	13	<0.05	1.597
PCSM	T4		0.66	10	<0.001	2.234
PICO	T4		0.68	17	<0.001	1.000
PILO	T4		0.23	15	<0.001	1.86
PIMO	T4		0.25	14	<0.05	1.000
PINI	T4		0.48	45	<0.001	1.217
PIPO2	T4		0.36	41	0.58	1.763
PIRE	T4		1.02	35	<0.001	3.897

Species Code	Type	Threshold(kPa)	Mean AI	*N*	***p-value***	edf
PISY3	T4		3.84	10	<0.1	1.000
PSMA	T4		0.39	24	<0.001	6.791
QUMA	T4		0.63	50	<0.001	3.652
QUPE	T4		1.78	36	<0.001	4.186
QURO	T4		1.15	76	<0.001	1
ABLA	T5		2.48	16	<0.001	1
AGAU	T5		0.87	29	<0.001	1.165
AUCH	T5		0.78	30	<0.001	2.126
LALY	T5		1.38	33	<0.005	1.003
LASI1	T5		0.62	19	<0.001	3.217
PIPN	T5		0.10	18	<0.001	4.373
PISY	T5		1.29	111	0.57	1.855
NOPU	T6		0.93	52	<0.001	5.952

Table S2: Climate data used in this study. Variables, data sources, spatial and temporal resolutions, and their specific applications in our analyses.

**Variables**	**Source**	**Spatial resolution**	**Temporal resolution**	**Analysis**
Maximum temperature	CRU	0.5°	Monthly	
Minimum temperature	CRU	0.5°	Monthly	VPD calculation
Actual vapour pressure	CRU	0.5°	Monthly	
Precipitation	CRU	0.5°	Monthly	GAMs, TRMs,<br>Aridity index calculation<br>Tree species classification
Potential evapotranspiration	CRU	0.5°	Monthly	Aridity index calculation
Shortwave radiation	Sheffield et al., 2006	0.5°	Monthly	GAMs, TRMs

Table S3: Earth system model used in this study, including the models, variables, scenarios, spatial and temporal resolutions, and time spanning.

Models	Variables	Scenarios	Nominal resolution	Temporal resolution	Time span
ACCESS-CM2	Maximum Temperature<br>Minimum Temperature<br>Relative humidity	SSP1-2.6<br>SSP2-4.5<br>SSP5-8.5	250 km (~2.25º)	Monthly	1901-2100
ACCESS-ESM1-5	Maximum Temperature<br>Minimum Temperature<br>Relative humidity	SSP1-2.6<br>SSP2-4.5<br>SSP5-8.5	250 km (~2.25º)	Monthly	1901-2100
AWI-CM-1-1-MR	Maximum Temperature<br>Minimum Temperature<br>Relative humidity	SSP1-2.6<br>SSP2-4.5<br>SSP5-8.5	100 km (~0.9º)	Monthly	1901-2100
CanESM5	Maximum Temperature<br>Minimum Temperature<br>Relative humidity	SSP1-2.6<br>SSP2-4.5<br>SSP5-8.5	500 km (~4.5º)	Monthly	1901-2100
CAS-ESM2-0	Maximum Temperature<br>Minimum Temperature<br>Relative humidity	SSP1-2.6<br>SSP2-4.5<br>SSP5-8.5	100 km (~0.9º)	Monthly	1901-2100
CMCC-ESM2	Maximum Temperature<br>Minimum Temperature<br>Relative humidity	SSP1-2.6<br>SSP2-4.5<br>SSP5-8.5	100 km (~0.9º)	Monthly	1901-2100
FIO-ESM-2-0	Maximum Temperature<br>Minimum Temperature<br>Relative humidity	SSP1-2.6<br>SSP2-4.5<br>SSP5-8.5	100 km (~0.9º)	Monthly	1901-2100

Models	Variables	Scenarios	Nominal resolution	Temporal resolution	Time span
	Maximum Temperature	SSP1-2.6			
MIROC6	Minimum Temperature	SSP2-4.5	250 km (~2.25º)	Monthly	1901-2100
	Relative humidity	SSP5-8.5			
	Maximum Temperature	SSP1-2.6			
MPI-ESM1-2-HR	Minimum Temperature	SSP2-4.5	100 km(~0.9º)	Monthly	1901-2100
	Relative humidity	SSP5-8.5			
	Maximum Temperature	SSP1-2.6			
MPI-ESM1-2-LR	Minimum Temperature	SSP2-4.5	250 km (~2.25º)	Monthly	1901-2100
	Relative humidity	SSP5-8.5			
	Maximum Temperature	SSP1-2.6			
NorESM2-M	Minimum Temperature	SSP2-4.5	250 km (~2.25º)	Monthly	1901-2100
	Relative humidity	SSP5-8.5			

Table S4: Criteria for classifying the six types of tree-growth responses to VPD based on the direction and significance of slopes before (k_1) and after (k_2) the breakpoint. Asterisks (*) indicate significant slopes, whereas "non-sig" indicates nonsignificant slopes.

k_1 \ k_2	**k_2>0***	**k_2 non-sig**	**k_2<0***
**k_1>0***	T5	T2	T1
**k_1 non-sig**	T5	T6	T3
**k_1<0***	T6	T4	T4

Supplementary Text1:

Linkages between exceedance of VPD thresholds and tree mortality

We compiled a global dataset of field-documented drought- and heat-related tree mortality events from peer-reviewed studies (1-6). In total, we assembled a tree mortality dataset comprising 1,613 documented tree mortality events that occurred between 1970 and 2018. Each mortality event includes the reported mortality calendar year, geographic location (latitude/longitude), and one or more reported dead species. To assess the association between VPD threshold exceedance and documented tree mortality, we treated each event–species combination as a mortality record and matched each record to a species group. First, the mortality record during 1970–2014 was required to have the same species code as the corresponding species group. Second, the mortality location was required to fall within the geographic extent (the convex hull constructed from all tree-ring sites within the group) represented by that species group (7,8). Event–species records outside the convex hull of the corresponding species group were not included in the threshold-exceedance analysis (Figs. S16 and S17). This procedure ensured that mortality events were compared with VPD thresholds estimated from tree-ring sites of the same species and from the same represented geographic region. Based on these criteria, 513 matched mortality event–species records were retained for subsequent analyses (Fig. S18). For each matched mortality event, we extracted the VPD conditions spanning from two years before to one year after the recorded mortality year using spatial bilinear interpolation.

Reference:

1.Caudullo G, & Barredo J I. (2019). A georeferenced dataset of drought and heat-induced tree mortality in Europe. *One Ecosystem, 4*, e37753.

2.Gazol A, & Camarero J J. (2022). Compound climate events increase tree drought mortality across European forests. *Science of the Total Environment, 816*, 151604.

3.Hammond W M, et al. (2022). Global field observations of tree die-off reveal hotter-drought fingerprint for Earth's forests. *Nature Communications, 13*(1), 1761.

4.Herguido E, et al. (2016). Contrasting growth and mortality responses to climate warming of two pine species in a continental Mediterranean ecosystem. *Forest ecology and management, 363*, 149-158.

5.Sánchez-Salguero R, & Camarero J J. (2020). Greater sensitivity to hotter droughts underlies juniper dieback and mortality in Mediterranean shrublands. *Science of the Total Environment, 721*, 137599.

6.Preisler Y, Tatarinov F, Grünzweig J M, & Yakir D. (2021). Seeking the "point of no return" in the sequence of events leading to mortality of mature trees. *Plant, cell & environment, 44*(5), 1315-1328.

7.Burgman M A, & Fox J C. (2003). *Bias in species range estimates from minimum convex polygons: implications for conservation and options for improved planning.* Paper presented at the Animal conservation forum.

8.Group R L T W. (2018). Mapping standards and data quality for the IUCN Red List Categories and Criteria. *Version, 1*(1.16), 30.
